# Incidence and management outcomes of chylothorax after pulmonary resection for primary lung cancer: a systematic review and meta-analysis

**DOI:** 10.3389/fonc.2026.1910973

**Published:** 2026-09-15

**Authors:** Taotao Zhang, Wenjing Kuang, Lan Zhang, Lijuan Du, Wenxi Zhang, Huiling Sun

**Affiliations:** 1 School of Medicine, Wuhan University of Science and Technology, Wuhan, China; 2 Department of General Surgery, General Hospital of Central Theater Command, Wuhan, China

**Keywords:** chylothorax, meta-analysis, non-surgical treatment, postoperative complication, primary lung cancer, pulmonary resection, systematic review

## Abstract

**Background:**

Chylothorax is an uncommon but clinically important complication of pulmonary resection for primary lung cancer and may complicate postoperative management and require treatment escalation. However, quantitative syntheses of its incidence and management outcomes are lacking. This meta-analysis pooled chylothorax incidence, the proportions of patients successfully treated non-surgically and receiving subsequent surgical or interventional treatment, and summarized clinical and management characteristics.

**Methods:**

Following PRISMA 2020, PubMed, Embase, Web of Science, Scopus, and the Cochrane Library were searched from inception to May 5, 2026. Original studies reporting incidence or management outcomes of postoperative chylothorax after pulmonary resection for primary lung cancer were included. Methodological quality was assessed with the JBI Critical Appraisal Checklist for Prevalence Studies. Random-effects single-proportion meta-analyses were performed. Exploratory subgroup analyses and meta-regression examined incidence; sensitivity analyses and assessments of small-study effects were conducted for the pooled outcomes.

**Results:**

Twenty-eight studies involving 212,637 patients were included, with 1,798 cases of postoperative chylothorax. The pooled incidence was 1.4% (95% CI: 1.0%–1.7%; I²=94.87%). Subgroup analyses by surgical approach and diagnostic criteria showed no significant between-subgroup differences, with high within-subgroup heterogeneity. Across 23 studies, the pooled proportion successfully treated non-surgically was 86.9% (95% CI: 82.0%–91.2%; I²=49.73%). Across 22 studies, the pooled proportion receiving subsequent surgical or interventional treatment was 12.8% (95% CI: 8.4%–17.9%; I²=52.32%). Leave-one-out and GLMM-based sensitivity analyses supported the robustness of the pooled estimates. Egger’s regression suggested a possible small-study effect for incidence, but trim-and-fill adjustment did not materially change the pooled estimate. Narrative synthesis showed variation in diagnostic criteria, initial non-surgical regimens, escalation strategies, and definitions and reporting of management outcomes.

**Conclusion:**

Chylothorax after pulmonary resection for primary lung cancer has a low incidence, but its management burden should not be overlooked. Most cases were controlled with initial non-surgical treatment, whereas some required subsequent surgical or interventional treatment. Diagnostic and treatment-escalation criteria remain inconsistent. Future studies should prespecify and validate diagnostic and treatment-escalation criteria and standardize the definition of successful non-surgical treatment and outcome reporting to improve comparability and provide more reliable evidence for postoperative surveillance and clinical management decisions.

**Systematic Review Registration:**

https://www.crd.york.ac.uk/PROSPERO/view/CRD420261392561, identifier CRD420261392561.

## 1 Introduction

Lung cancer is one of the malignancies with the greatest global burden in terms of incidence and mortality, and pulmonary resection remains an important treatment modality for resectable primary lung cancer (1, 2). Chylothorax is an uncommon but clinically important complication after pulmonary resection and results from the abnormal accumulation of chyle-rich lymphatic fluid in the pleural cavity; postoperatively, it is most often associated with injury to the thoracic duct or its tributaries (3–5). Persistent chyle leakage can result in ongoing losses of lipids, proteins, lymphocytes, immunoglobulins, and electrolytes, potentially leading to malnutrition, impaired immune function, fluid and electrolyte disturbances, and prolonged chest drainage and hospital stay (3, 4, 6). Clinical management of chylothorax may involve chest drainage, dietary modification and nutritional support, pleurodesis, and, when necessary, surgical or interventional treatment, thereby increasing the complexity of perioperative management and treatment burden (4–7). Therefore, systematically evaluating the incidence and management outcomes of chylothorax after pulmonary resection for primary lung cancer may provide a more comprehensive understanding of its clinical burden and inform optimization of postoperative surveillance and treatment decision-making.

A previous systematic review synthesized evidence on chylothorax after cardiothoracic surgery. The 2023 systematic review by Santos et al. included multiple types of cardiothoracic surgery, including esophagectomy, cardiac surgery, aortic surgery, pulmonary resection, and mediastinal surgery, and performed meta-analyses of postoperative chylothorax incidence and outcomes related to non-surgical management (8). Although some studies of pulmonary resection were included, the study population was not restricted to patients with primary lung cancer, and chylothorax incidence and management outcomes were not separately pooled for this specific population. Moreover, dedicated quantitative syntheses of the proportion of patients successfully treated non-surgically and the proportion receiving subsequent surgical or interventional treatment after pulmonary resection for primary lung cancer remain lacking. Given that pulmonary resection for primary lung cancer commonly involves hilar and mediastinal lymph node evaluation or dissection, and that chylothorax is closely associated with injury to relevant lymphatic pathways during these procedures (5, 9), a focused evaluation of chylothorax incidence and management outcomes in this specific population is warranted.

Accordingly, this systematic review and meta-analysis quantitatively pooled the incidence of chylothorax after pulmonary resection for primary lung cancer, the proportion of patients successfully treated non-surgically, and the proportion receiving subsequent surgical or interventional treatment; further explored potential sources of heterogeneity in incidence; and systematically summarized the diagnostic criteria, management approaches, and other management-related clinical outcomes reported across studies. The aim was to provide a more comprehensive synthesis of the available evidence on postoperative chylothorax in this population and more targeted information to support clinical assessment and management.

## 2 Methods

### 2.1 Study design and registration

This systematic review and meta-analysis was reported in accordance with the Preferred Reporting Items for Systematic Reviews and Meta-Analyses (PRISMA 2020) statement (10). The study protocol was prospectively registered in PROSPERO under registration number CRD420261392561.

### 2.2 Search strategy

PubMed, Embase, Web of Science, Scopus, and the Cochrane Library were systematically searched from database inception to May 5, 2026. The search strategy was developed around three core concepts: “lung cancer,” “pulmonary resection,” and “chylothorax,” and was adapted to the subject headings, search fields, and syntax of each database. The complete search strategies are provided in **Supplementary Table S3**.

### 2.3 Inclusion and exclusion criteria

Studies were included if they met the following criteria: (1) the study population comprised patients with primary lung cancer who underwent pulmonary resection; (2) the study reported at least one of the following outcomes and provided extractable data: postoperative chylothorax incidence or clinical management outcomes; and (3) studies involving mixed populations were included only when data for patients with primary lung cancer could be extracted separately.

Studies were excluded if they met any of the following criteria: (1) non-original clinical studies, conference abstracts, basic or animal studies, or case reports; (2) studies primarily focused on the prevention, diagnosis, or treatment techniques for chylothorax and from which data on the target outcomes of this review could not be extracted; or (3) studies for which the full text was unavailable, key data could not be extracted, or a clearly defined denominator for the relevant outcome was lacking, as well as duplicate publications or less informative reports from overlapping cohorts.

### 2.4 Study selection and data extraction

Search results were imported into EndNote 21 for reference management and deduplication. Two reviewers independently screened titles and abstracts and assessed the full texts of potentially eligible studies to determine whether they met the inclusion criteria. Any disagreements during the screening process were resolved through discussion, with a third reviewer consulted when necessary.

A predefined standardized data extraction form was used to extract information from the included studies. Data were independently extracted by two reviewers and cross-checked after extraction; any disagreements were resolved through discussion, with a third reviewer consulted when necessary. Extracted information included first author, year of publication, country or region, study design, study period, study population, surgical approach and extent of resection, lymph node management, diagnostic criteria for chylothorax, number of postoperative chylothorax cases, and the corresponding total sample size. For management outcomes, the main data extracted included the sample size included in analyses of chylothorax management outcomes, non-surgical treatment strategies, number of patients successfully treated non-surgically, number of patients receiving subsequent surgical or interventional treatment, final treatment outcomes, and mortality. Other relevant outcomes reflecting the management course and clinical burden of chylothorax were also extracted.

Successful non-surgical treatment was primarily defined as control of chylothorax after non-surgical treatment without subsequent surgical or interventional treatment. Subsequent surgical or interventional treatment was primarily defined as surgical or interventional treatment administered after initial non-surgical treatment. Studies in which it was unclear whether surgical or interventional treatment occurred after initial non-surgical treatment were summarized narratively and excluded from the quantitative synthesis of this outcome. If the number of patients included differed across management outcomes within the same study, the sample size reported for the corresponding outcome in the original study was used for the corresponding analysis.

### 2.5 Methodological quality assessment

The methodological quality of the included studies was assessed using the Joanna Briggs Institute (JBI) Critical Appraisal Checklist for Prevalence Studies (11). Two reviewers independently performed the appraisal. Disagreements were resolved through discussion, with a third reviewer consulted when necessary. Each item was rated as “Yes,” “No,” “Unclear,” or “Not applicable.” Based on the number of items rated “Yes,” studies were classified as high quality (7–9 items), moderate quality (4–6 items), or low quality (0–3 items).

### 2.6 Statistical analysis

Single-proportion meta-analyses were performed to pool the incidence of postoperative chylothorax, the proportion of patients successfully treated non-surgically, and the proportion receiving subsequent surgical or interventional treatment. Given the potential clinical and methodological heterogeneity across the included studies, random-effects models were used for the primary analyses, with between-study variance estimated using the DerSimonian–Laird method (12). Because some outcome proportions were close to 0 or 1 and zero-event studies were present, the Freeman–Tukey double-arcsine transformation was applied to stabilize the variance of proportions (13). Pooled results were expressed as proportions with 95% confidence intervals (CIs).

Between-study heterogeneity was assessed using Cochran’s Q test and the I² statistic (14). For chylothorax incidence, exploratory subgroup analyses were conducted according to surgical approach and diagnostic criteria for chylothorax. Surgical approach was classified into three groups: predominantly or exclusively minimally invasive surgery, open or mixed surgery, and surgical approach not reported or unclear. Diagnostic criteria were classified into three groups: criteria explicitly including a triglyceride threshold, other reported diagnostic criteria, and diagnostic criteria not reported or unclear. Differences between subgroups were assessed using the Q test for subgroup differences. In addition, univariable meta-regression was performed using the natural logarithm of sample size as a covariate to explore potential sources of heterogeneity.

Leave-one-out sensitivity analyses were performed to assess whether the pooled estimates were materially influenced by any individual study. To evaluate the robustness of the pooled results across different statistical models, additional sensitivity analyses were conducted using a random-effects generalized linear mixed model (GLMM) with a binomial distribution and logit link, based on the number of events and corresponding sample size in each study (15, 16). The pooled proportions and 95% CIs obtained from the GLMM were compared with those from the primary analyses using the Freeman–Tukey double-arcsine transformation and the DerSimonian–Laird estimator for between-study variance to determine whether the study conclusions changed materially with the choice of statistical model.

Funnel plots were visually inspected, and Egger’s regression test was used to assess small-study effects (17). When Egger’s test was statistically significant, the trim-and-fill method was further applied as an exploratory analysis (18). Management-related clinical outcomes that could not be quantitatively pooled were summarized narratively. All statistical analyses were performed using Stata version 18.0, and a two-sided P value <0.05 was considered statistically significant.

## 3 Results

### 3.1 Characteristics of the included studies

A total of 732 records were identified through the database searches. After deduplication, title and abstract screening, and full-text eligibility assessment, 28 studies were ultimately included in the systematic review and meta-analysis (5, 6, 19–44). The detailed study selection process is shown in **Figure 1**. **Table 1** summarizes the basic characteristics of the included studies, including study region, study design, study population, diagnostic criteria for chylothorax, and incidence. Complete study characteristics are provided in **Supplementary Table S1**.

**Figure 1:**
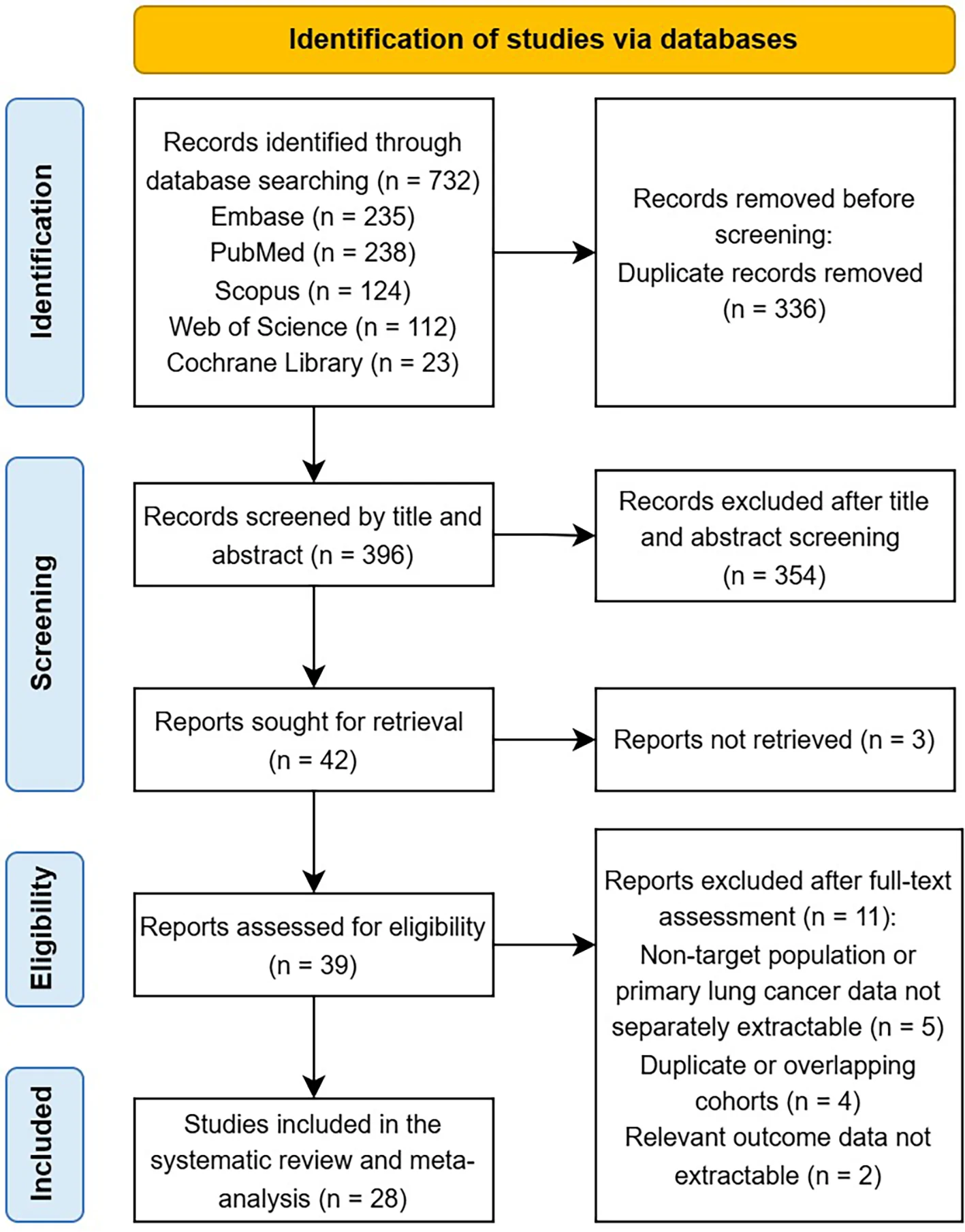
PRISMA flow diagram of the study selection process.

**Table 1:** Characteristics of the included studies and incidence of chylothorax after pulmonary resection for primary lung cancer.

Study	Country /region	Study design and period	Population	Definition of chylothorax	Chylothorax incidence, n/N (%)
Akin et al. (2012) (19)	Turkey	R, SC; 2000.01–2010.06	NSCLC patients undergoing lung resection	Biochemical confirmation after clinical suspicion; pleural fluid TG > 110 mg/dL	26/1389 (1.87)
Bollen et al. (1993) (20)	Netherlands	R, SC, HC; 1986–1991	NSCLC patients undergoing lung resection	NR	2/155 (1.29)
Bryant et al. (2014) (21)	USA	R, SC; 2000.01–2012.12	Lung cancer patients undergoing lung resection	Chest tube drainage fluid TG > 110 mg/dL	41/2838 (1.44)
Cho et al. (2014) (22)	South Korea	R, SC; 2000.01–2010.12	Primary lung cancer patients undergoing lung resection	Biochemical analysis of pleural fluid after clinical suspicion; pleural fluid TG > 110 mg/dL	67/3120 (2.15)
Dong et al. (2022) (23)	China	R, SC; 2012.01–2020.01	Primary lung cancer patients undergoing lung resection	Biochemical analysis or chyle testing after clinical suspicion; pleural fluid TG > 110 mg/dL or positive Sudan III staining	37/12515 (0.30)
Fang et al. (2019) (24)	China	R, SC; 2015.05–2018.05	Lung cancer patients undergoing lung resection	Increased chest drainage volume or a change in drainage appearance; positive chyle test	22/1305 (1.69)
Haruki et al. (2021) (25)	Japan	R, SC; 2011.01–2020.04	Primary lung cancer patients undergoing lung resection	NR	3/390 (0.77)
Ji et al. (2024) (26)	China	R, SC; 2021.03–2022.12	Primary lung cancer patients undergoing lung resection	White or turbid chest tube drainage after resumption of a regular diet; pleural fluid TG > 1.24 mmol/L (> 110 mg/dL)	108/2942 (3.67)
Kamtam et al. (2026) (27)	USA	R, MC; 2015.01–2024.06	Patients with primary NSCLC undergoing elective pulmonary resection	STS definition: chylous pleural effusion requiring treatment intervention within 30 d	1110/152823 (0.73)
Kanzaki et al. (2003) (28)	Japan	R, SC; 1987.07–2001.12	Primary lung cancer patients undergoing lung resection	Pleural fluid analysis showing chylomicrons; the fluid clarified after ether shaking	6/941 (0.64)
Kutlu et al. (2003) (29)	Turkey	R, SC; 1990–2000	NSCLC patients undergoing lung resection	Typical chylous drainage after oral intake; biochemical criteria: specific gravity > 1.012, pleural fluid protein > 50% of plasma, microscopic fat globules, and TG > 500 mg/dL	7/673 (1.04)
Le Pimpec-Barthes et al. (2002) (30)	France	R, MC; 1981.03–2001.06	Lung cancer patients undergoing lung resection	Milky pleural effusion; pleural fluid TG > 100 mg/dL, lymphocytes > 90%, and protein concentration close to that of plasma	25/3834 (0.65)
Li et al. (2025) (31)	China	R, SC; 2009–2022	Patients with small-sized (≤2 cm) NSCLC undergoing surgical resection	NR	20/2713 (0.74)
Liu et al. (2014) (32)	Taiwan, China	R, SC; 2010.01–2013.08	Primary NSCLC patients undergoing lung resection	Chylous chest tube drainage; confirmed by pleural fluid TG > 110 mg/dL	20/776 (2.58)
Liu et al. (2022) (33)	China	R, SC; 2012.06–2020.09	Primary lung cancer patients undergoing lobectomy (PSM cohort)	Increased chest drainage volume with milky appearance after resumption of a regular diet; positive chyle test (Sudan III staining)	24/868 (2.76)
Petrella et al. (2020) (34)	Italy	R, SC; 1998.01–2018.12	Patients with pulmonary malignancies undergoing anatomic lung resection	Chylous appearance of chest drainage; confirmed by pleural fluid TG > 110 mg/dL	30/5072 (0.59)
Shimizu et al. (2002) (35)	Japan	R, SC; 1992.07–2000.02	Lung cancer patients undergoing lung resection	Chylous chest tube drainage; confirmed by drainage fluid TG > 110 mg/dL and chylomicrons	27/1110 (2.43)
Son et al. (2025) (6)	South Korea	R, SC; 2009.09–2024.08	Lung cancer patients undergoing lung resection	Chylous or turbid chest tube drainage after fat intake; pleural fluid TG ≥ 110 mg/dL, or TG 50–110 mg/dL with lymphocyte proportion ≥ 80%	21/1038 (2.02)
Takuwa et al. (2013) (36)	Japan	R, SC; 2003.10–2010.03	Primary lung cancer patients undergoing lung resection	Chylous chest tube drainage; confirmed by drainage fluid TG > 110 mg/dL	37/1580 (2.34)
Uchida et al. (2016) (37)	Japan	R, SC; 2008.01–2012.09	Primary lung cancer patients undergoing lung resection	Milky pleural effusion; confirmed by drainage fluid TG > 110 mg/dL	50/1235 (4.05)
Wan et al. (2009) (38)	China	R, SC; 2001.09–2008.12	Lung cancer patients undergoing lung resection	Pleural effusion or increased chest drainage after resumption of a regular diet, with a white, turbid appearance; positive pleural fluid chyle test	17/1472 (1.15)
Wang et al. (2014) (39)	China	R, MC; 2001.01–2008.01	NSCLC patients undergoing lobectomy	NR	14/5378 (0.26)
Wang et al. (2019) (40)	China	P, SC; 2016.09–2017.03	Primary NSCLC patients undergoing lung resection	High-volume pleural effusion after resumption of a regular diet, with pleural fluid changing from serous to milky yellow	4/108 (3.70)
Watanabe et al. (2005) (41)	Japan	R, SC; 1997–2004	Patients with clinical stage I primary lung cancer undergoing lung resection	NR	7/411 (1.70)
Yang et al. (2013) (42)	China	R, SC; 2007.05–2012.05	NSCLC patients undergoing lobectomy	NR	3/264 (1.14)
Yang et al. (2022) (43)	China	R, SC; 2016.01–2021.12	NSCLC patients undergoing lung resection	True chylothorax: chest drainage fluid was milky white, pale red, or pale yellow and turbid; pleural fluid TG > 6.1 mmol/L, positive chyle test	21/1584 (1.33)
Yasuura et al. (2022) (5)	Japan	R, SC; 2002.09–2018.03	Primary lung cancer patients undergoing lung resection	Chest drainage fluid markedly white and turbid, or pleural effusion TG > 110 mg/dL	37/2019 (1.83)
Zhao et al. (2003) (44)	China	R, SC; since 1985, end time NR	Lung cancer patients undergoing lung resection	Clinical manifestations and pleural fluid characteristics; Sudan III staining as supportive evidence	12/4084 (0.29)

R, retrospective; P, prospective; SC, single-center; MC, multicenter; HC, historical control; NSCLC, non-small cell lung cancer; PSM, propensity score matching; STS, Society of Thoracic Surgeons; TG, triglyceride; NR, not reported; d, days.

The included studies were published between 1993 and 2026 and were conducted mainly in Asia, Europe, and North America. With the exception of one prospective study, all remaining studies were retrospective, and most were single-center studies. Across the 28 studies, a total of 212,637 patients with primary lung cancer who underwent pulmonary resection were included, of whom 1,798 developed postoperative chylothorax. The diagnostic criteria for chylothorax varied across studies, and some studies did not clearly report specific diagnostic criteria.

The methodological quality of the included studies was assessed using the JBI Critical Appraisal Checklist for Prevalence Studies. Twenty studies were classified as high quality, eight as moderate quality, and none as low quality. The complete appraisal results are provided in **Supplementary Table S2**.

### 3.2 Incidence of postoperative chylothorax

The primary random-effects analysis based on 28 studies showed that the pooled incidence of chylothorax after pulmonary resection for primary lung cancer was 1.4% (95% CI: 1.0%–1.7%). Between-study heterogeneity was high (I²=94.87%, Q(27)=526.44, P<0.001). The forest plot for chylothorax incidence is shown in **Figure 2**.

**Figure 2:**
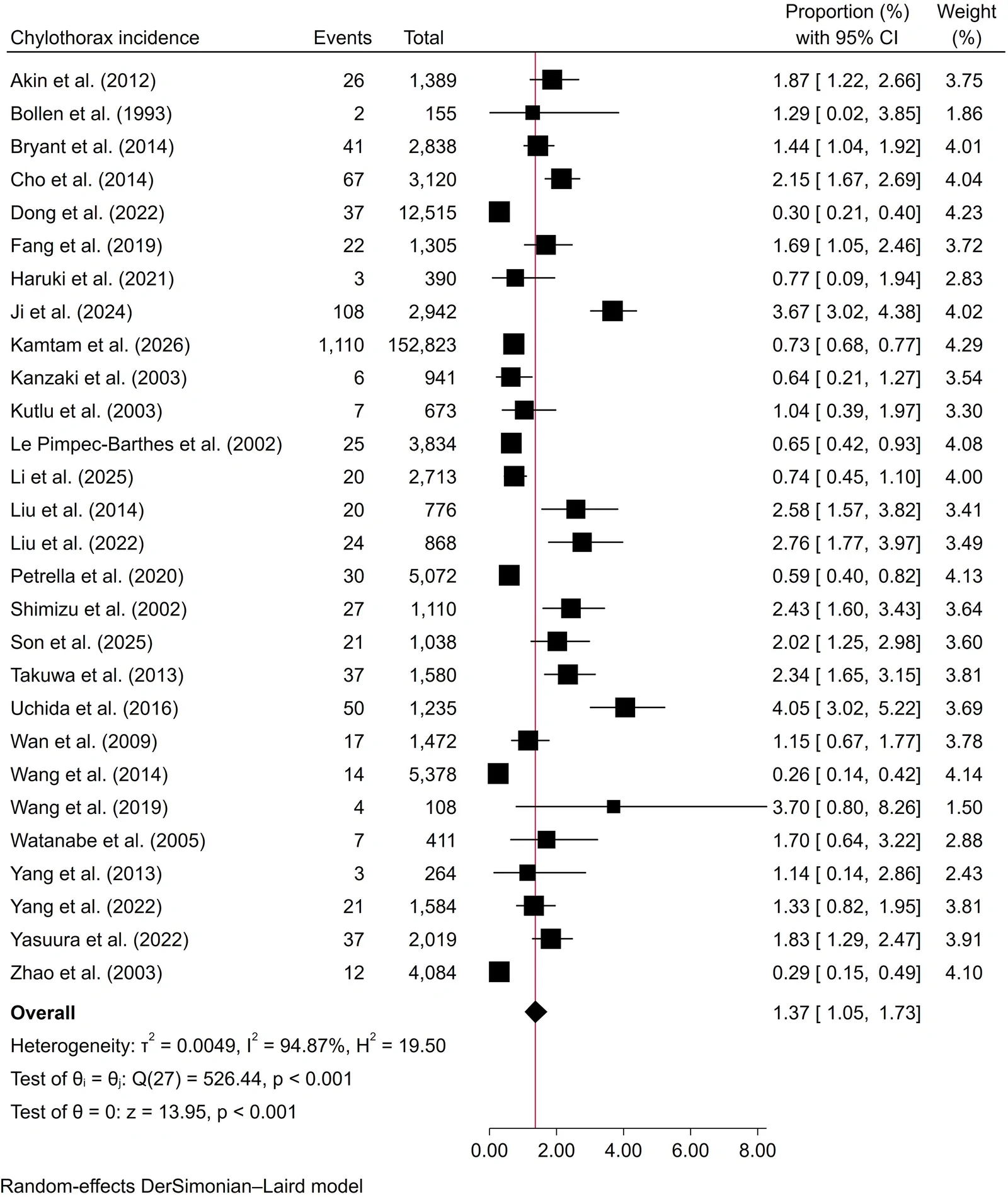
Forest plot of the incidence of postoperative chylothorax.

### 3.3 Subgroup analyses and meta-regression of incidence

Given the high between-study heterogeneity observed in the overall incidence analysis, subgroup analyses were further conducted according to surgical approach and diagnostic criteria for chylothorax, together with univariable meta-regression using the natural logarithm of sample size as a covariate. When stratified by surgical approach, the pooled incidences were 1.6% (95% CI: 1.0%–2.4%; I²=96.52%) in the predominantly or exclusively minimally invasive surgery subgroup, 1.0% (95% CI: 0.3%–2.0%; I²=90.96%) in the open or mixed surgery subgroup, and 1.4% (95% CI: 0.9%–2.0%; I²=93.45%) in the subgroup in which surgical approach was not reported or unclear. The between-subgroup difference was not statistically significant (Q_β_(2)=1.18, P = 0.556), and heterogeneity remained high within each subgroup, indicating that this stratification by surgical approach did not adequately explain the overall heterogeneity. When stratified by diagnostic criteria, the pooled incidences were 1.7% (95% CI: 1.1%–2.5%; I²=96.27%) for studies with diagnostic criteria explicitly including a triglyceride threshold, 1.1% (95% CI: 0.6%–1.6%; I²=89.91%) for studies using other reported diagnostic criteria, and 0.8% (95% CI: 0.3%–1.4%; I²=77.48%) for studies in which the diagnostic criteria were not reported or unclear. The between-subgroup difference was not statistically significant (Q_β_(2)=4.12, P = 0.128), and heterogeneity remained high within the subgroups, indicating that stratification by diagnostic criteria did not adequately explain the overall heterogeneity. Forest plots for the two subgroup analyses are provided in **Supplementary Figures S1, S2**.

Univariable meta-regression showed a negative association between the natural logarithm of sample size and the estimated incidence of chylothorax (β=−0.029, 95% CI: −0.054 to −0.004, P = 0.022), indicating that larger studies tended to report lower incidence estimates. Although this association was statistically significant, residual heterogeneity in the meta-regression model remained high (I²=94.04%), with R²=0.00%, indicating that sample size did not explain the observed between-study heterogeneity in this model.

### 3.4 Management outcomes of postoperative chylothorax

For management outcomes, the proportions of patients successfully treated non-surgically and those receiving subsequent surgical or interventional treatment were quantitatively pooled separately. The meta-analysis of successful non-surgical treatment included 23 studies. The primary random-effects analysis showed that the pooled proportion of patients successfully treated non-surgically was 86.9% (95% CI: 82.0%–91.2%), with moderate between-study heterogeneity (I²=49.73%, Q(22)=43.76, P = 0.004). The corresponding forest plot is shown in **Figure 3**.

**Figure 3:**
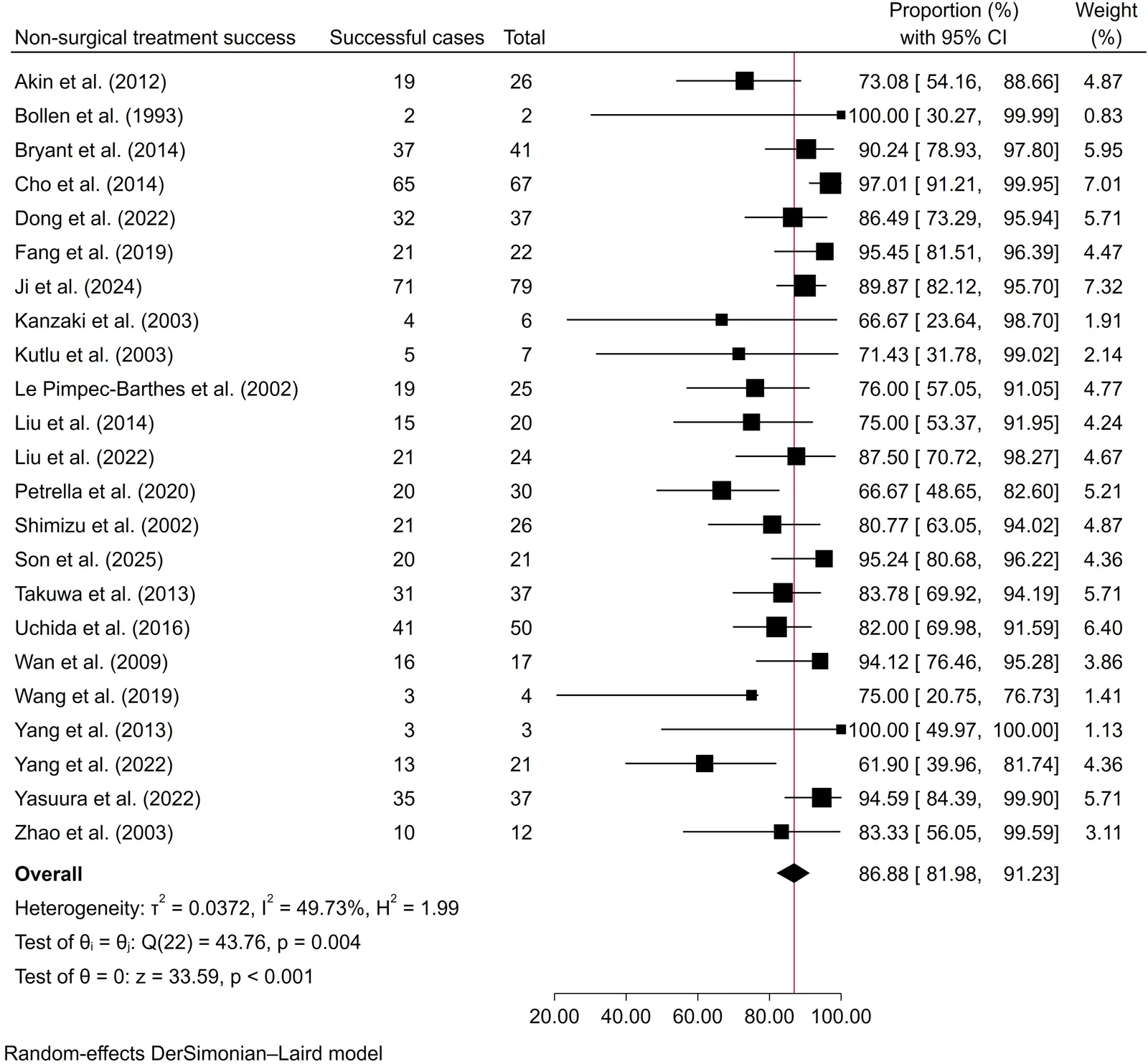
Forest plot of the proportion of patients successfully treated non-surgically among patients with postoperative chylothorax.

A total of 24 studies reported surgical or interventional treatment. In two studies, it was unclear whether surgical or interventional treatment was performed after initial non-surgical treatment; these studies were therefore summarized narratively only. The remaining 22 studies were included in the quantitative synthesis. The random-effects model yielded a pooled proportion of patients receiving subsequent surgical or interventional treatment of 12.8% (95% CI: 8.4%–17.9%), with moderate between-study heterogeneity (I²=52.32%, Q(21)=44.04, P = 0.002). The corresponding forest plot is shown in **Figure 4**. Because the two meta-analyses did not include exactly the same studies and the two pooled proportions were estimated independently, the two proportions should not be interpreted as strictly complementary.

**Figure 4:**
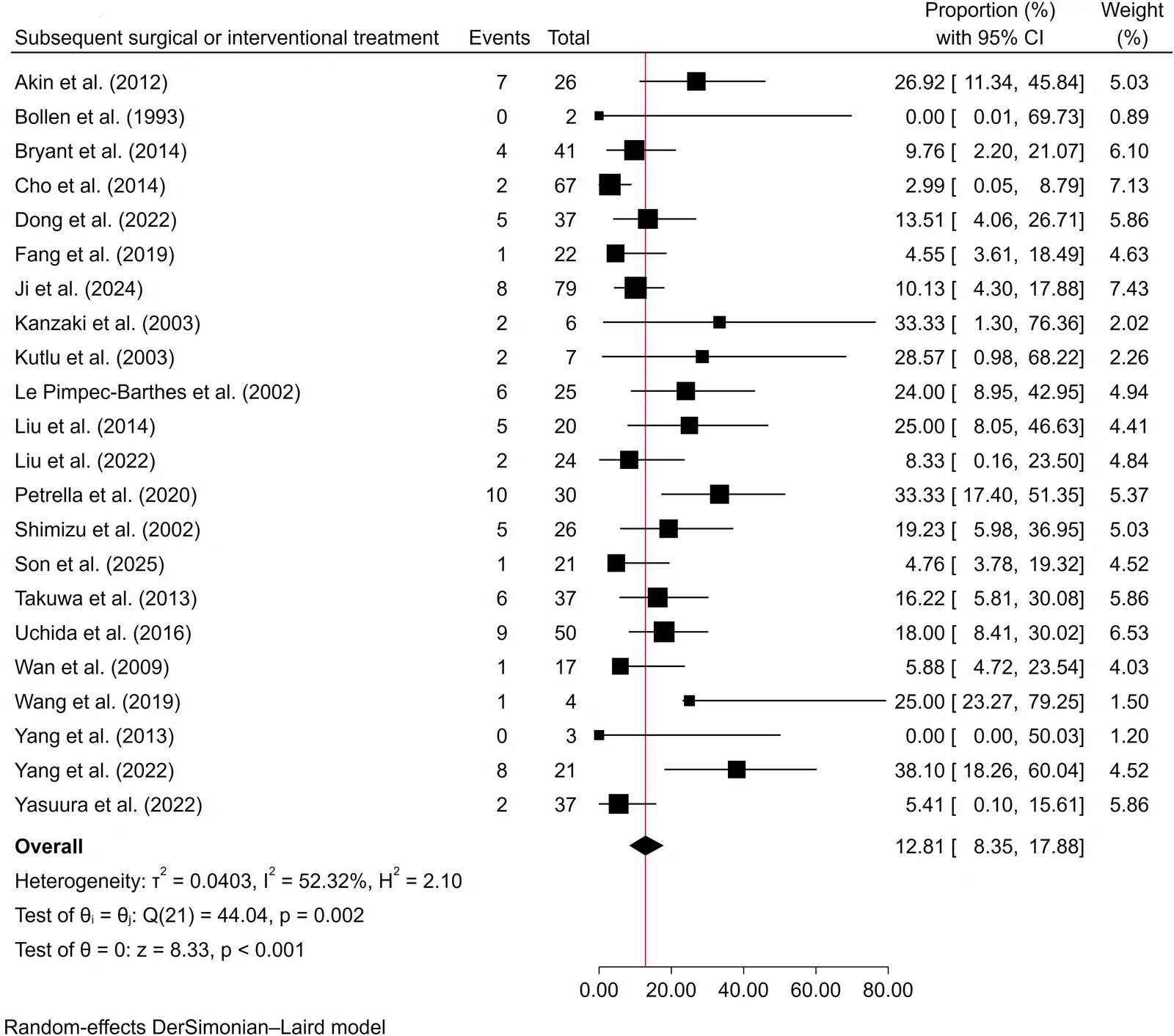
Forest plot of the proportion of patients receiving subsequent surgical or interventional treatment among patients with postoperative chylothorax.

In addition to the quantitatively pooled outcomes described above, some studies reported other outcomes, including final control of chylothorax, mortality, duration of chest tube drainage, length of hospital stay, drainage volume, and time to resumption of a normal diet. Among studies reporting final treatment outcomes, most cases of chylothorax were controlled with non-surgical treatment or, when necessary, after subsequent surgical or interventional treatment. Mortality events were generally infrequent among studies reporting mortality outcomes. Because these outcomes varied considerably in their definitions, reporting methods, and statistical formats, they were not quantitatively pooled and were summarized narratively only. Detailed results are presented in **Table 2**.

**Table 2:** Main management outcomes of postoperative chylothorax after pulmonary resection for primary lung cancer.

Study	No. of cases analyzed for management outcomes, n	Non-surgical treatment, n/N	Non-surgical treatment success, n/N (%)	Surgical or interventional treatment, n/N (%)	Final treatment outcome and mortality
Akin et al. (2012) (19)	26	26/26; NPO + TPN, supplemented by talc pleurodesis via chest tube	19/26 (73.1)	Surgery: 7/26 (26.9); reoperation via thoracotomy, leak suturing or en bloc ligation	Chylothorax control: 25/26 (96.2); mortality: 1/26 (3.8), due to sepsis and multiple organ failure
Bollen et al. (1993) (20)	2	2/2; specific regimen NR	2/2 (100.0)	Surgery: 0/2 (0.0)	Mortality: 0/2 (0.0)
Bryant et al. (2014) (21)	41	41/41; NPO + subcutaneous somatostatin, switched to MCT diet after meeting criteria	37/41 (90.2)	Surgery: 4/41 (9.8); pleurodesis and clipping of lymphatic leak area	Surgery-related mortality: 0/41 (0.0)
Cho et al. (2014) (22)	67	67/67; NPO + TPN (46 cases) or low-LCT diet (21 cases); pleurodesis for dietary treatment failure	65/67 (97.0)	Surgery: 2/67 (3.0); procedure NR	Pleurodesis success: 27/27; pleurodesis-related deaths: 0 cases
Dong et al. (2022) (23)	37	37/37; NPO + TPN + continuous closed thoracic drainage, albumin infusion if necessary; intrathoracic pleurodesis for ineffective conservative treatment	32/37 (86.5)	Surgery: 5/37 (13.5); VATS thoracic duct ligation	Chylothorax control in the surgery group: 5/5 (100.0)
Fang et al. (2019) (24)	22	22/22; low-fat, high-carbohydrate, high-protein diet, with maintained chest drainage; intravenous nutrition, albumin, somatostatin, electrolyte supplementation, and intrathoracic pleurodesis if necessary	21/22 (95.5)	Surgery: 1/22 (4.5); thoracoscopic ligation of the main thoracic duct	Chylothorax control: 22/22 (100.0); mortality: 0/22 (0.0)
Ji et al. (2024) (26)	108 (including 79 in the low-fat diet group)	108/108; low-fat diet in 79 cases (fat ≤ 5 g/d), TPN in 29 cases	71/79 (89.9)	Surgery: 8/79 (10.1; low-fat diet group); procedure NR	Mortality: 0/79 (0.0; low-fat diet group); recurrence after reoperation: 0/8 (0.0)
Kamtam et al. (2026) (27)	1110	NR	NR	Surgery: 237/1110 (21.4); procedure NR	30-d operative mortality: 15 cases (1.4%)
Kanzaki et al. (2003) (28)	6	6/6; continuous chest drainage, NPO, central venous nutrition (CVN); MCT diet added in 3 cases	4/6 (66.7)	Surgery: 2/6 (33.3); VATS ligation of the injured thoracic duct or branches	Chylothorax control: 6/6 (100.0); in-hospital mortality: 0/6 (0.0)
Kutlu et al. (2003) (29)	7	7/7; NPO + TPN, switched to a fat-free diet on postoperative day 7 if leakage volume was < 500 mL/d	5/7 (71.4)	Surgery: 2/7 (28.6); re-thoracotomy, fistula ligation reinforced with surrounding tissue	Chylothorax control: 7/7 (100.0); mortality: 1/7 (14.3), due to pneumonia
Le Pimpec-Barthes et al. (2002) (30)	25	25/25; chest tube drainage or thoracentesis; fat restriction with MCT substitution, with maintenance of nutrition and electrolyte balance	19/25 (76.0)	Surgery: 6/25 (24.0); thoracic duct ligation, leak suturing, or fibrin glue	Mortality: 1/25 (4.0), due to ARDS
Liu et al. (2014) (32)	20	20/20; NPO + parenteral nutrition; some patients received a fat-free/low-fat diet	15/20 (75.0)	Surgery: 5/20 (25.0); VATS reoperation, leak clipping or thoracic duct ligation, with pleurodesis in some cases	Chylothorax control: 20/20 (100.0); mortality: 0/20 (0.0)
Liu et al. (2022) (33)	24	24/24; initial low-fat, high-protein diet; chest tube clamping, chemical pleurodesis, or octreotide if necessary	21/24 (87.5)	Surgery: 2/24 (8.3); thoracic duct suturing/ligation	Chylothorax control: 23/24 (95.8); mortality: 1/24 (4.2), due to cardiopulmonary failure
Petrella et al. (2020) (34)	30	30/30; NPO + TPN; switched to a low-fat diet (fat < 10 g/d) after drainage became clear for ≥ 24 h and drainage volume was < 800 mL/d	20/30 (66.7)	Surgery: 10/30 (33.3); thoracic duct ligation, with possible clipping of lymphatic leak point	Chylothorax control: 30/30 (100.0); 30-d mortality: 1/30 (3.3), due to postoperative acute hemorrhagic pseudocolytis
Shimizu et al. (2002) (35)	26 (treatment-strategy analysis cohort; 27 cases occurred in total)	26/26; NPO + TPN; OK-432 pleurodesis if necessary	21/26 (80.8)	Surgery: 5/26 (19.2); procedure NR	Chylothorax control: 26/26 (100.0); mortality: 0/26 (0.0)
Son et al. (2025) (6)	21	21/21; NPO + TPN (24–48 h), then switched to a fat-free/low-fat diet; pleurodesis if necessary	20/21 (95.2)	Surgery: 1/21 (4.8); thoracic duct ligation	30-d mortality: 0/21 (0.0)
Takuwa et al. (2013) (36)	37	37/37; low-fat diet (fat < 10 g/d); OK-432 pleurodesis if necessary	31/37 (83.8)	Surgery: 6/37 (16.2); video-assisted thoracic duct ligation, with leak suturing/TachoComb coverage in some cases	Chylothorax control: 37/37 (100.0); 30-d and in-hospital mortality: 0/37 (0.0)
Uchida et al. (2016) (37)	50	50/50; low-fat diet (fat < 20 g/d); NPO or OK-432 pleurodesis if necessary	41/50 (82.0)	Surgery: 9/50 (18.0); ligation of thoracic duct leak point or en bloc ligation	Chylothorax control: 50/50 (100.0); 30-d and in-hospital mortality: 0/50 (0.0)
Wan et al. (2009) (38)	17	17/17; low-fat, high-carbohydrate, high-protein diet; chest drainage; intravenous nutritional support; intrathoracic Sapylin injection	16/17 (94.1)	Surgery: 1/17 (5.9); thoracic re-entry through the original incision, with ligation of the proximal and distal ends of the fistula	Chylothorax control: 17/17 (100.0)
Wang et al. (2019) (40)	4	4/4; specific regimen NR	3/4 (75.0)	Surgery: 1/4 (25.0); VATS thoracic duct ligation	Mortality: 0/4 (0.0)
Yang et al. (2013) (42)	3	3/3; specific regimen NR	3/3 (100.0)	Surgery: 0/3 (0.0)	Chylothorax control: 3/3 (100.0)
Yang et al. (2022) (43)	21 (true chylothorax)	21/21; conservative treatment, with adequate drainage, nutritional support, and pleurodesis in 13/21	13/21 (61.9)	Surgery: 8/21 (38.1); right-sided VATS thoracic duct clipping	NR
Yasuura et al. (2022) (5)	37	37/37; low-fat diet (fat restriction 10–30 g/d); OK-432 pleurodesis if necessary (5/37)	35/37 (94.6)	Surgery: 2/37 (5.4); supradiaphragmatic thoracic duct ligation with TachoComb coverage of the leak point	Chylothorax control: 37/37 (100.0); 30-d mortality: 0/37 (0.0)
Zhao et al. (2003) (44)	12	10/12; thoracentesis drainage or prolonged chest tube retention	10/12 (83.3)	Surgery: 2/12 (16.7); thoracic duct ligation	Chylothorax control: 12/12 (100.0)

This table summarizes the main management outcomes. Studies without extractable management outcomes are not shown. When denominators differed by outcome, the denominator used is reported in the corresponding cell. Detailed treatment strategies and additional clinical outcomes are provided in **Supplementary Table S4**. Kamtam et al. (27) and Zhao et al. (44) were summarized descriptively but were excluded from the pooled analysis of subsequent surgical or interventional treatment because the treatment sequence was not clearly reported.

NR, not reported; NPO, nil per os/no oral intake; TPN, total parenteral nutrition; CVN, central venous nutrition; MCT, medium-chain triglyceride; LCT, long-chain triglyceride; VATS, video-assisted thoracoscopic surgery; OK-432, Picibanil; ARDS, acute respiratory distress syndrome; TachoComb, fibrinogen-coated collagen patch/hemostatic material; d, days.

### 3.5 Sensitivity analyses and assessment of small-study effects

Leave-one-out sensitivity analyses showed that, after excluding any individual study, the pooled estimates of the incidence of chylothorax after pulmonary resection for primary lung cancer, the proportion of patients successfully treated non-surgically, and the proportion receiving subsequent surgical or interventional treatment remained stable. Specifically, the pooled incidence of chylothorax ranged from 1.3% to 1.4%, the proportion of patients successfully treated non-surgically ranged from 85.9% to 88.0%, and the proportion receiving subsequent surgical or interventional treatment ranged from 11.7% to 13.8%. These results indicated that none of the main pooled estimates was materially influenced by any individual study. The sensitivity analysis results are shown in **Supplementary Figures S3–S5**.

Alternative-model sensitivity analyses were conducted using a random-effects GLMM with a binomial distribution and logit link. The pooled incidence of postoperative chylothorax, the proportion of patients successfully treated non-surgically, and the proportion receiving subsequent surgical or interventional treatment were 1.2% (95% CI: 0.9%–1.6%), 85.5% (95% CI: 81.1%–89.9%), and 14.2% (95% CI: 10.2%–19.3%), respectively. These estimates were generally close to the corresponding primary-analysis estimates of 1.4% (95% CI: 1.0%–1.7%), 86.9% (95% CI: 82.0%–91.2%), and 12.8% (95% CI: 8.4%–17.9%), indicating that the main study conclusions did not change materially after using an alternative statistical model.

Funnel plots were visually assessed, and Egger’s regression test was used to evaluate small-study effects. For the analysis of chylothorax incidence, Egger’s regression test suggested a possible small-study effect (P = 0.025). The trim-and-fill analysis estimated and imputed seven potentially missing studies, and the adjusted pooled incidence did not change materially. Egger’s regression tests for the proportion of patients successfully treated non-surgically and the proportion receiving subsequent surgical or interventional treatment showed no statistical evidence of small-study effects (P = 0.199 and 0.231, respectively). Funnel plots are shown in **Supplementary Figures S6–S8**.

## 4 Discussion

This study systematically synthesized the incidence and management outcomes of chylothorax after pulmonary resection for primary lung cancer. The results showed that the overall incidence of postoperative chylothorax was low. Among patients who developed chylothorax, most were controlled with initial non-surgical treatment, although some subsequently required surgical or interventional treatment. Given the differences across the included studies in diagnostic criteria, non-surgical treatment regimens, criteria for treatment escalation, and the definitions and reporting of management outcomes, interpretation of these pooled estimates should take full account of the clinical and methodological heterogeneity across studies.

The incidence of postoperative chylothorax showed high between-study heterogeneity. Subgroup analyses by surgical approach and diagnostic criteria did not adequately explain this heterogeneity. Although meta-regression showed a negative association between sample size and incidence estimates, it did not explain the between-study variance. Previous studies have suggested that pathologic N2 status, surgical side, and the extent and intensity of mediastinal lymph node dissection may be associated with the risk of postoperative chylothorax (7, 21, 45). In addition, the included studies differed in patient composition, surgical era, and center-specific perioperative management practices, which may have influenced the occurrence, recognition, or reporting of chylothorax. Because these factors were inconsistently or insufficiently reported in the original studies, they could not be quantitatively analyzed in this study. Consequently, the strength or relative importance of their associations with the risk of postoperative chylothorax could not be determined, nor could their contributions to the overall heterogeneity be further quantified. In this context, the pooled incidence in this study should be interpreted as an average estimate across different clinical and methodological settings rather than as a fixed level of risk applicable to all centers, patient populations, or specific surgical procedures.

For management outcomes, this study showed that the pooled proportion of patients successfully treated non-surgically was 86.9%, while the pooled proportion receiving subsequent surgical or interventional treatment was 12.8%. Because the two analyses did not include exactly the same studies and were conducted separately, these proportions should not be regarded as strictly complementary. The included studies reported management strategies including low-fat or medium-chain triglyceride diets, fasting, parenteral nutrition, chest drainage, somatostatin and its analogues, pleurodesis, and surgical or interventional treatment when necessary. The initial non-surgical regimens, subsequent escalation strategies, and criteria for determining treatment success and escalation varied across studies, limiting the comparability of management outcomes across studies. Therefore, the pooled success proportion of 86.9% should be interpreted as an overall estimate across different non-surgical management strategies and cannot be attributed to any specific intervention or standardized regimen. The 12.8% pooled proportion receiving subsequent surgical or interventional treatment should likewise be interpreted cautiously in the context of inconsistent escalation criteria across studies. Because the available studies lack direct comparisons between different treatment strategies, the relative effectiveness of different management approaches cannot yet be determined, nor can these findings be used to conclude that non-surgical treatment is superior to early surgical or interventional treatment.

Leave-one-out sensitivity analyses showed that, after excluding any individual study, the pooled estimates of chylothorax incidence, the proportion of patients successfully treated non-surgically, and the proportion receiving subsequent surgical or interventional treatment remained stable, indicating that the main results were not materially influenced by any individual study. The three pooled estimates obtained from the GLMM alternative-model sensitivity analyses were generally close to those from the primary analyses, indicating that the main study conclusions did not change materially after changing the statistical model. It should be noted that these two types of sensitivity analyses were used only to assess the robustness of the pooled results and could not explain the sources of the high heterogeneity observed in the chylothorax incidence analysis. Egger’s regression test for chylothorax incidence suggested a possible small-study effect. Although the pooled incidence did not change materially after trim-and-fill adjustment, funnel plot asymmetry and the results of Egger’s test may also have been influenced by between-study heterogeneity and other factors. Therefore, the interpretation of small-study effects and potential publication bias should remain cautious, and the current evidence is insufficient to directly conclude that publication bias is present.

From a clinical practice perspective, based on the findings of this study and the available evidence, a stepwise management approach centered on early recognition, initial non-surgical management, and dynamic assessment may be considered for chylothorax after pulmonary resection for primary lung cancer. Postoperatively, chylothorax should be suspected when chest drainage output persistently increases without an obvious cause or remains high, particularly when the drainage fluid becomes cloudy or chylous after oral intake is resumed (46, 47). Diagnosis should incorporate the triglyceride level in pleural drainage fluid, with further testing for chylomicrons when necessary, rather than relying solely on the appearance of the drainage fluid (3, 48). When the patient’s overall condition permits, non-surgical treatment may be considered as the initial management option, with the main goals of maintaining effective pleural drainage, reducing chyle production, and maintaining nutritional status and fluid and electrolyte balance (4, 6). During treatment, chyle output and its trend should be continuously monitored, together with the duration of chyle leakage, nutritional status, and fluid and electrolyte balance. If chyle output remains persistently high, shows no downward trend after initial treatment, or the patient’s nutritional status deteriorates, the need for surgical or interventional treatment should be assessed promptly (6, 7, 49). Given that the available evidence remains insufficient to establish a uniform threshold for chyle output or a standardized observation period for non-surgical treatment, decisions regarding further surgical or interventional treatment should not be based solely on a single threshold or a predetermined time limit. Instead, chyle output and its trend, response to initial treatment, nutritional status, and the patient’s overall clinical condition should be considered comprehensively. This stepwise management approach may inform clinical decision-making but should not yet be regarded as a validated, standardized management algorithm.

This study has several limitations. Most of the included studies were retrospective single-center studies and may therefore have been affected by selection bias, information bias, and differences in management practices across centers. Although subgroup analyses were conducted according to surgical approach and diagnostic criteria, and meta-regression was performed using the natural logarithm of sample size as a covariate, the high heterogeneity observed in the chylothorax incidence analysis was not fully explained. Potentially influential factors, including patient composition, surgical side, characteristics of lymph node management, surgical era, and perioperative management practices, were inconsistently or insufficiently reported in the original studies, precluding further quantification of their contributions to heterogeneity. In addition, non-surgical treatment regimens and definitions of management outcomes varied across studies, limiting comparability across studies and clinical interpretation. Definitions and reporting formats also differed for outcomes such as duration of chest tube drainage, length of hospital stay, chyle output, time to resumption of a normal diet, and nutrition-related indicators; therefore, these outcomes could not be quantitatively pooled. The available studies also lack direct comparisons among non-surgical, surgical, and interventional treatments; therefore, the relative effectiveness of different treatment strategies cannot yet be determined, and uniform criteria and timing for treatment escalation remain difficult to establish.

Future studies should prespecify criteria for the diagnosis of chylothorax, success and failure of non-surgical treatment, and treatment escalation in their study protocols, validate the applicability of these criteria, and standardize the definitions and reporting of key management outcomes. In addition, greater emphasis should be placed on the collection and reporting of the timing of chyle leakage, chyle output and its trends, surgical and lymph node-related characteristics, treatment interventions and responses, as well as the duration of chest tube drainage, length of hospital stay, nutritional status, and mortality outcomes, to improve comparability across studies. When sample size permits, the incidence of chylothorax should also be reported stratified by clinical and surgical characteristics, and management outcomes should be reported stratified by chyle output and its trends and by treatment strategy to explore potential sources of between-study heterogeneity. Meanwhile, prospective multicenter studies are needed to compare the clinical effectiveness of different treatment strategies and to clarify the indications and timing for treatment escalation. The stepwise management approach summarized in this review also requires evaluation in prospective studies to determine its feasibility and impact on clinical outcomes.

## Data Availability

The original contributions presented in the study are included in the article/**Supplementary Material**. Further inquiries can be directed to the corresponding author.
