# Supplementary material for: Incidence and management outcomes of chylothorax after pulmonary resection for primary lung cancer: a systematic review and meta-analysis
Source: Front Oncol. 2026 Sep 15;16:1910973. doi: 10.3389/fonc.2026.1910973 (PMC13619438; doi:10.3389/fonc.2026.1910973)
Supplement: Supplementary file 1 [file DataSheet1.pdf]

Supplementary Table S1. Complete characteristics of the included studies and incidence of chylothorax after pulmonary resection for primary lung cancer

Study	Country /region	Study design and period	Population	Surgical approach and extent of resection	Lymph node management	Definition of chylothorax	Chylothorax incidence, n/N (%)
Akin et al. (2012) (19)	Turkey	R, SC; 2000.01–2010.06	NSCLC patients undergoing lung resection	Approach: NR; extent of resection: NR	NR	Biochemical confirmation after clinical suspicion; pleural fluid TG > 110 mg/dL	26/1389 (1.87)
Bollen et al. (1993) (20)	Netherlands	R, SC, HC; 1986–1991	NSCLC patients undergoing lung resection	Approach: thoracotomy; extent of resection: pneumonectomy, lobectomy, sleeve lobectomy, segmentectomy, or wedge resection	Mediastinal lymph node dissection or systematic sampling; only suspicious lymph nodes were sampled in the control group	NR	2/155 (1.29)
Bryant et al. (2014) (21)	USA	R, SC; 2000.01–2012.12	Lung cancer patients undergoing lung resection	Approach: RATS or thoracotomy; extent of resection: lobectomy or segmentectomy/wedge resection	Complete mediastinal lymph node dissection	Chest tube drainage fluid TG > 110 mg/dL	41/2838 (1.44)
Cho et al. (2014) (22)	South Korea	R, SC; 2000.01–2010.12	Primary lung cancer patients undergoing lung resection	Approach: NR; extent of resection: NR	Mediastinal lymph node dissection	Biochemical analysis of pleural fluid after clinical suspicion; pleural fluid TG > 110 mg/dL	67/3120 (2.15)
Dong et al. (2022) (23)	China	R, SC; 2012.01–2020.01	Primary lung cancer patients undergoing lung resection	Approach: VATS; extent of resection: NR	NR	Biochemical analysis or chyle testing after clinical suspicion; pleural fluid TG > 110 mg/dL or positive Sudan III staining	37/12515 (0.30)
Fang et al. (2019) (24)	China	R, SC; 2015.05–2018.05	Lung cancer patients undergoing lung resection	Approach: VATS; extent of resection: NR	NR	Increased chest drainage volume or a change in drainage appearance; positive chyle test	22/1305 (1.69)
Haruki et al. (2021) (25)	Japan	R, SC; 2011.01–2020.04	Primary lung cancer patients undergoing lung resection	Approach: VATS or RATS; extent of resection: lobectomy	Systematic or lobe-specific mediastinal lymph node dissection	NR	3/390 (0.77)
Ji et al. (2024) (26)	China	R, SC; 2021.03–2022.12	Primary lung cancer patients undergoing lung resection	Approach: VATS or RATS; extent of resection: lung resection (specific extent NR)	Lymph node dissection	White or turbid chest tube drainage after resumption of a regular diet; pleural fluid TG > 1.24 mmol/L (> 110 mg/dL)	108/2942 (3.67)
Kamtam et al. (2026) (27)	USA	R, MC; 2015.01–2024.06	Patients with primary NSCLC undergoing elective pulmonary resection	Approach: predominantly VATS or robotic, with some open procedures; extent of resection: wedge resection, segmentectomy, lobectomy, bilobectomy, or pneumonectomy	Lymph node dissection or sampling; extent quantified by total LN count	STS definition: chylous pleural effusion requiring treatment intervention within 30 d	1110/152823 (0.73)
Kanzaki et al. (2003) (28)	Japan	R, SC; 1987.07–2001.12	Primary lung cancer patients undergoing lung resection	Approach: NR; extent of resection: lobectomy	Systematic mediastinal lymph node dissection	Pleural fluid analysis showing chylomicrons; the fluid clarified after ether shaking	6/941 (0.64)
Kutlu et al. (2003) (29)	Turkey	R, SC; 1990–2000	NSCLC patients undergoing lung resection	Approach: thoracotomy; extent of resection: lobectomy or pneumonectomy	Mediastinal lymph node sampling or radical dissection	Typical chylous drainage after oral intake; biochemical criteria: specific gravity > 1.012, pleural fluid protein > 50% of plasma, microscopic fat globules, and TG > 500 mg/dL	7/673 (1.04)
Le Pimpec-Barthes et al. (2002) (30)	France	R, MC; 1981.03–2001.06	Lung cancer patients undergoing lung resection	Approach: NR; extent of resection: NR	NR	Milky pleural effusion; pleural fluid TG > 100 mg/dL, lymphocytes > 90%, and protein concentration close to that of plasma	25/3834 (0.65)
Li et al. (2025) (31)	China	R, SC; 2009–2022	Patients with small-sized (≤2 cm) NSCLC undergoing surgical resection	Approach: NR; extent of resection: wedge resection, segmentectomy, or lobectomy	No lymph node evaluation, or lymph node evaluation with selective or systematic sampling/dissection	NR	20/2713 (0.74)
Liu et al. (2014) (32)	Taiwan, China	R, SC; 2010.01–2013.08	Primary NSCLC patients undergoing lung resection	Approach: VATS; extent of resection: lung resection (specific extent NR)	Routine mediastinal lymph node dissection	Chylous chest tube drainage; confirmed by pleural fluid TG > 110 mg/dL	20/776 (2.58)

Study	Country /region	Study design and period	Population	Surgical approach and extent of resection	Lymph node management	Definition of chylothorax	Chylothorax incidence, n/N (%)
Liu et al. (2022) (33)	China	R, SC; 2012.06–2020.09	Primary lung cancer patients undergoing lobectomy (PSM cohort)	Approach: RATS or VATS; extent of resection: lobectomy	Systematic lymph node dissection	Increased chest drainage volume with milky appearance after resumption of a regular diet; positive chyle test (Sudan III staining)	24/868 (2.76)
Petrella et al. (2020) (34)	Italy	R, SC; 1998.01–2018.12	Patients with pulmonary malignancies undergoing anatomic lung resection	Approach: NR; extent of resection: anatomic lung resection (specific extent NR)	Radical mediastinal lymph node dissection	Chylous appearance of chest drainage; confirmed by pleural fluid TG > 110 mg/dL	30/5072 (0.59)
Shimizu et al. (2002) (35)	Japan	R, SC; 1992.07–2000.02	Lung cancer patients undergoing lung resection	Approach: NR; extent of resection: lobectomy or more extensive resection	Systematic mediastinal lymph node dissection	Chylous chest tube drainage; confirmed by drainage fluid TG > 110 mg/dL and chylomicrons	27/1110 (2.43)
Son et al. (2025) (6)	South Korea	R, SC; 2009.09–2024.08	Lung cancer patients undergoing lung resection	Approach: MVATS, SVATS, or RATS; extent of resection: segmentectomy, lobectomy, or bilobectomy	Lymph node dissection	Chylous or turbid chest tube drainage after fat intake; pleural fluid TG ≥ 110 mg/dL, or TG 50–110 mg/dL with lymphocyte proportion ≥ 80%	21/1038 (2.02)
Takuwa et al. (2013) (36)	Japan	R, SC; 2003.10–2010.03	Primary lung cancer patients undergoing lung resection	Approach: NR; extent of resection: lobectomy or more extensive resection	Systematic mediastinal lymph node dissection	Chylous chest tube drainage; confirmed by drainage fluid TG > 110 mg/dL	37/1580 (2.34)
Uchida et al. (2016) (37)	Japan	R, SC; 2008.01–2012.09	Primary lung cancer patients undergoing lung resection	Approach: NR; extent of resection: anatomic lung resection (specific extent NR)	NR	Milky pleural effusion; confirmed by drainage fluid TG > 110 mg/dL	50/1235 (4.05)
Wan et al. (2009) (38)	China	R, SC; 2001.09–2008.12	Lung cancer patients undergoing lung resection	Approach: NR; extent of resection: lobectomy, bilobectomy, or pneumonectomy	Complete mediastinal lymph node dissection	Pleural effusion or increased chest drainage after resumption of a regular diet, with a white, turbid appearance; positive pleural fluid chyle test	17/1472 (1.15)
Wang et al. (2014) (39)	China	R, MC; 2001.01–2008.01	NSCLC patients undergoing lobectomy	Approach: VATS or thoracotomy; extent of resection: lobectomy	Systematic lymph node dissection	NR	14/5378 (0.26)
Wang et al. (2019) (40)	China	P, SC; 2016.09–2017.03	Primary NSCLC patients undergoing lung resection	Approach: mainly VATS, with some conversions to thoracotomy; extent of resection: segmentectomy, lobectomy, bilobectomy, lobectomy combined with segmentectomy, or pneumonectomy	Mediastinal and hilar lymph node dissection	High-volume pleural effusion after resumption of a regular diet, with pleural fluid changing from serous to milky yellow	4/108 (3.70)
Watanabe et al. (2005) (41)	Japan	R, SC; 1997–2004	Patients with clinical stage I primary lung cancer undergoing lung resection	Approach: VATS or thoracotomy; extent of resection: segmentectomy, lobectomy, bilobectomy, or pneumonectomy	Systematic lymph node dissection	NR	7/411 (1.70)
Yang et al. (2013)(42)	China	R, SC; 2007.05–2012.05	NSCLC patients undergoing lobectomy	Approach: VATS; extent of resection: lobectomy	Systematic lymph node dissection	NR	3/264 (1.14)
Yang et al. (2022) (43)	China	R, SC; 2016.01–2021.12	NSCLC patients undergoing lung resection	Approach: NR; extent of resection: lobectomy or sublobar resection	Lymph node dissection	True chylothorax: chest drainage fluid was milky white, pale red, or pale yellow and turbid; pleural fluid TG > 6.1 mmol/L, positive chyle test	21/1584 (1.33)
Yasuura et al. (2022) (5)	Japan	R, SC; 2002.09–2018.03	Primary lung cancer patients undergoing lung resection	Approach: NR; extent of resection: lobectomy, bilobectomy, or pneumonectomy	Mediastinal lymph node dissection	Chest drainage fluid markedly white and turbid, or pleural effusion TG > 110 mg/dL	37/2019 (1.83)
Zhao et al. (2003) (44)	China	R, SC; since 1985, end time NR	Lung cancer patients undergoing lung resection	Approach: NR; extent of resection: lung resection (specific extent NR)	NR	Clinical manifestations and pleural fluid characteristics; Sudan III staining as supportive evidence	12/4084 (0.29)

Abbreviations: R = retrospective; P = prospective; SC = single-center; MC = multicenter; HC = historical control; NSCLC = non-small cell lung cancer; PSM = propensity score matching; VATS = video-assisted thoracoscopic surgery; RATS = robot-assisted thoracic surgery; MVATS = multi-port video-assisted thoracoscopic surgery; SVATS = single-port video-assisted thoracoscopic surgery; LN = lymph node; STS = Society of Thoracic Surgeons; TG = triglyceride; NR = not reported; d = days.

Supplementary Table S2. Methodological quality assessment of the included studies using the JBI checklist

Study	Q1	Q2	Q3	Q4	Q5	Q6	Q7	Q8	Q9	Total score
Akin et al. (2012) (19)	Y	Y	Y	Y	Y	Y	Y	Y	U	8
Bollen et al. (1993) (20)	Y	U	U	Y	Y	U	U	Y	U	4
Bryant et al. (2014) (21)	Y	Y	Y	Y	Y	Y	Y	Y	Y	9
Cho et al. (2014) (22)	Y	Y	Y	Y	Y	Y	Y	Y	U	8
Dong et al. (2022) (23)	Y	Y	Y	Y	Y	Y	Y	Y	U	8
Fang et al. (2019) (24)	Y	U	Y	Y	Y	Y	Y	Y	U	7
Haruki et al. (2021) (25)	Y	Y	U	Y	Y	U	U	Y	U	5
Ji et al. (2024) (26)	Y	Y	Y	Y	Y	Y	Y	Y	U	8
Kamtam et al. (2026) (27)	Y	Y	Y	Y	Y	Y	Y	Y	Y	9
Kanzaki et al. (2003) (28)	Y	Y	Y	Y	Y	Y	Y	Y	U	8
Kutlu et al. (2003) (29)	Y	Y	Y	Y	Y	Y	Y	Y	U	8
Le Pimpec-Barthes et al. (2002) (30)	Y	Y	Y	Y	Y	Y	Y	Y	U	8
Li et al. (2025) (31)	Y	Y	Y	Y	Y	U	U	Y	U	6
Liu et al. (2014) (32)	Y	Y	Y	Y	Y	Y	Y	Y	U	8
Liu et al. (2022) (33)	Y	Y	Y	Y	Y	Y	Y	Y	U	8
Petrella et al. (2020) (34)	Y	Y	Y	Y	Y	Y	Y	Y	Y	9
Shimizu et al. (2002) (35)	Y	Y	Y	Y	Y	Y	Y	Y	U	8
Son et al. (2025) (6)	Y	Y	Y	Y	Y	Y	Y	Y	Y	9
Takuwa et al. (2013) (36)	Y	Y	Y	Y	Y	Y	Y	Y	Y	9
Uchida et al. (2016) (37)	Y	Y	Y	Y	Y	Y	Y	Y	U	8
Wan et al. (2009) (38)	Y	Y	Y	Y	Y	Y	Y	Y	U	8
Wang et al. (2014) (39)	Y	Y	Y	Y	Y	U	U	Y	U	6
Wang et al. (2019) (40)	Y	Y	U	Y	Y	U	U	Y	Y	6
Watanabe et al. (2005) (41)	Y	Y	U	Y	Y	U	U	Y	U	5
Yang et al. (2013) (42)	Y	Y	U	Y	Y	U	U	Y	U	5
Yang et al. (2022) (43)	Y	Y	Y	Y	Y	Y	Y	Y	U	8
Yasuura et al. (2022) (5)	Y	Y	Y	Y	Y	Y	Y	Y	U	8
Zhao et al. (2003) (44)	Y	Y	Y	Y	Y	U	U	Y	U	6

Note: The JBI checklist for studies reporting prevalence data includes nine items: Q1, appropriate sample frame; Q2, appropriate participant recruitment; Q3, adequate sample size; Q4, detailed description of study subjects and setting; Q5, sufficient coverage of the identified sample; Q6, valid methods for identifying the condition; Q7, standard and reliable measurement of the condition; Q8, appropriate statistical analysis; and Q9, adequate response rate or appropriate management of a low response rate. The total score was calculated as the number of items rated "Y". Study quality was classified as high (7–9), moderate (4–6), or low (0–3).

Abbreviations: JBI = Joanna Briggs Institute; Y = yes; U = unclear.

Supplementary Table S3. Complete search strategies for each database

Search date: May 5, 2026. Searches were conducted from database inception to May 5, 2026, and the strategies are presented as run in each database.

**PubMed**
**Stepwise search strategy:**<br><br>#1 "Lung Neoplasms"[Mesh] OR "lung cancer"[Title/Abstract] OR "lung cancers"[Title/Abstract] OR "lung carcinoma"[Title/Abstract] OR "lung carcinomas"[Title/Abstract] OR "lung neoplasm"[Title/Abstract] OR "lung neoplasms"[Title/Abstract] OR "lung tumor"[Title/Abstract] OR "lung tumors"[Title/Abstract] OR "lung tumour"[Title/Abstract] OR "lung tumours"[Title/Abstract] OR "pulmonary carcinoma"[Title/Abstract] OR "pulmonary carcinomas"[Title/Abstract] OR "pulmonary neoplasm"[Title/Abstract] OR "pulmonary neoplasms"[Title/Abstract] OR "pulmonary tumor"[Title/Abstract] OR "pulmonary tumors"[Title/Abstract] OR "pulmonary tumour"[Title/Abstract] OR "pulmonary tumours"[Title/Abstract] OR "non-small cell lung cancer"[Title/Abstract] OR "non-small-cell lung cancer"[Title/Abstract] OR NSCLC[Title/Abstract] OR "small cell lung cancer"[Title/Abstract] OR "small-cell lung cancer"[Title/Abstract] OR SCLC[Title/Abstract] OR "lung adenocarcinoma"[Title/Abstract] OR "lung squamous cell carcinoma"[Title/Abstract]<br><br>#2 "Chylothorax"[Mesh] OR chylothorax[Title/Abstract] OR chylothoraces[Title/Abstract] OR "chylous pleural effusion"[Title/Abstract] OR "chylous effusion"[Title/Abstract] OR "chyle leak"[Title/Abstract] OR "chyle leakage"[Title/Abstract] OR "chylous leak"[Title/Abstract] OR "chylous leakage"[Title/Abstract] OR "chylous fistula"[Title/Abstract] OR "chyle fistula"[Title/Abstract] OR chylorrhea[Title/Abstract] OR chylorrhoea[Title/Abstract] OR "thoracic duct injury"[Title/Abstract] OR "thoracic duct injuries"[Title/Abstract] OR "thoracic duct leak"[Title/Abstract] OR "thoracic duct leakage"[Title/Abstract] OR "thoracic duct fistula"[Title/Abstract] OR "lymphatic leak"[Title/Abstract] OR "lymphatic leakage"[Title/Abstract] OR "lymphatic fistula"[Title/Abstract]<br><br>#3 "Pulmonary Surgical Procedures"[Mesh] OR "Pneumonectomy"[Mesh] OR "Thoracic Surgery, Video-Assisted"[Mesh] OR lobectomy[Title/Abstract] OR lobectomies[Title/Abstract] OR "pulmonary lobectomy"[Title/Abstract] OR "lung lobectomy"[Title/Abstract] OR pneumonectomy[Title/Abstract] OR pneumonectomies[Title/Abstract] OR segmentectomy[Title/Abstract] OR segmentectomies[Title/Abstract] OR "wedge resection"[Title/Abstract] OR "wedge resections"[Title/Abstract] OR resection[Title/Abstract] OR resections[Title/Abstract] OR "radical resection"[Title/Abstract] OR "radical resections"[Title/Abstract] OR "lung resection"[Title/Abstract] OR "lung resections"[Title/Abstract] OR "pulmonary resection"[Title/Abstract] OR "pulmonary resections"[Title/Abstract] OR "lung surgery"[Title/Abstract] OR "pulmonary surgery"[Title/Abstract] OR thoracotomy[Title/Abstract] OR thoracotomies[Title/Abstract] OR thoracoscopic[Title/Abstract] OR thoracoscopy[Title/Abstract] OR VATS[Title/Abstract] OR robotic[Title/Abstract] OR "robot-assisted"[Title/Abstract] OR "robotic-assisted"[Title/Abstract] OR RATS[Title/Abstract] OR "lymph node dissection"[Title/Abstract] OR "lymph node dissections"[Title/Abstract] OR "mediastinal lymph node dissection"[Title/Abstract] OR "mediastinal lymph node dissections"[Title/Abstract] OR "mediastinal lymphadenectomy"[Title/Abstract] OR lymphadenectomy[Title/Abstract] OR "lymph node sampling"[Title/Abstract]<br><br>#4 #1 AND #2 AND #3<br><br>**Records retrieved (n):<u>238</u>**

**Embase**
**Stepwise search strategy:**<br><br>#1 'lung cancer':ti,ab,kw OR 'lung cancers':ti,ab,kw OR 'lung carcinoma':ti,ab,kw OR 'lung carcinomas':ti,ab,kw OR 'lung neoplasm':ti,ab,kw OR 'lung neoplasms':ti,ab,kw OR 'lung tumor':ti,ab,kw OR 'lung tumors':ti,ab,kw OR 'lung tumour':ti,ab,kw OR 'lung tumours':ti,ab,kw OR 'pulmonary carcinoma':ti,ab,kw OR 'pulmonary carcinomas':ti,ab,kw OR 'pulmonary neoplasm':ti,ab,kw OR 'pulmonary neoplasms':ti,ab,kw OR 'pulmonary tumor':ti,ab,kw OR 'pulmonary tumors':ti,ab,kw OR 'pulmonary tumour':ti,ab,kw OR 'pulmonary tumours':ti,ab,kw OR 'non-small cell lung cancer':ti,ab,kw OR 'non-small-cell lung cancer':ti,ab,kw OR nsclc:ti,ab,kw OR 'small cell lung cancer':ti,ab,kw OR 'small-cell lung cancer':ti,ab,kw OR sclc:ti,ab,kw OR 'lung adenocarcinoma':ti,ab,kw OR 'lung squamous cell carcinoma':ti,ab,kw<br><br>#2 chylothorax:ti,ab,kw OR chylothoraces:ti,ab,kw OR 'chylous pleural effusion':ti,ab,kw OR 'chylous effusion':ti,ab,kw OR 'chyle leak':ti,ab,kw OR 'chyle leakage':ti,ab,kw OR 'chylous leak':ti,ab,kw OR 'chylous leakage':ti,ab,kw OR 'chylous fistula':ti,ab,kw OR 'chyle fistula':ti,ab,kw OR chylorrhea:ti,ab,kw OR chylorrhoea:ti,ab,kw OR 'thoracic duct injury':ti,ab,kw OR 'thoracic duct injuries':ti,ab,kw OR 'thoracic duct leak':ti,ab,kw OR 'thoracic duct leakage':ti,ab,kw OR 'thoracic duct fistula':ti,ab,kw<br><br>#3 lobectomy:ti,ab,kw OR lobectomies:ti,ab,kw OR 'pulmonary lobectomy':ti,ab,kw OR 'lung lobectomy':ti,ab,kw OR pneumonectomy:ti,ab,kw OR pneumonectomies:ti,ab,kw OR segmentectomy:ti,ab,kw OR segmentectomies:ti,ab,kw OR 'wedge

resection':ti,ab,kw OR 'wedge resections':ti,ab,kw OR 'lung resection':ti,ab,kw OR 'lung resections':ti,ab,kw OR 'pulmonary resection':ti,ab,kw OR 'pulmonary resections':ti,ab,kw OR 'lung surgery':ti,ab,kw OR 'pulmonary surgery':ti,ab,kw OR thoracotomy:ti,ab,kw OR thoracotomies:ti,ab,kw OR thoracoscopic:ti,ab,kw OR thoracoscopy:ti,ab,kw OR vats:ti,ab,kw OR robotic:ti,ab,kw OR 'robot-assisted':ti,ab,kw OR 'robotic-assisted':ti,ab,kw OR rats:ti,ab,kw OR 'lymph node dissection':ti,ab,kw OR 'lymph node dissections':ti,ab,kw OR 'mediastinal lymph node dissection':ti,ab,kw OR 'mediastinal lymph node dissections':ti,ab,kw OR 'mediastinal lymphadenectomy':ti,ab,kw OR lymphadenectomy:ti,ab,kw OR 'lymph node sampling':ti,ab,kw

#4 #1 AND #2 AND #3

Records retrieved (n):235

Web of Science

Stepwise search strategy:

#1 Topic=("lung cancer" OR "lung cancers" OR "lung carcinoma" OR "lung carcinomas" OR "lung neoplasm" OR "lung neoplasms" OR "lung tumor" OR "lung tumors" OR "lung tumour" OR "lung tumours" OR "pulmonary carcinoma" OR "pulmonary carcinomas" OR "pulmonary neoplasm" OR "pulmonary neoplasms" OR "pulmonary tumor" OR "pulmonary tumors" OR "pulmonary tumour" OR "pulmonary tumours" OR "non-small cell lung cancer" OR "non-small-cell lung cancer" OR NSCLC OR "small cell lung cancer" OR "small-cell lung cancer" OR SCLC OR "lung adenocarcinoma" OR "lung squamous cell carcinoma")

#2 Topic=(chylothorax OR chylothoraces OR "chylous pleural effusion" OR "chylous effusion" OR "chyle leak" OR "chyle leakage" OR "chylous leak" OR "chylous leakage" OR "chylous fistula" OR "chyle fistula" OR chylorrhea OR chylorrhoea OR "thoracic duct injury" OR "thoracic duct injuries" OR "thoracic duct leak" OR "thoracic duct leakage" OR "thoracic duct fistula" OR "lymphatic leak" OR "lymphatic leakage" OR "lymphatic fistula")

#3 Topic=(lobectomy OR lobectomies OR "pulmonary lobectomy" OR "lung lobectomy" OR pneumonectomy OR pneumonectomies OR segmentectomy OR segmentectomies OR "wedge resection" OR "wedge resections" OR resection OR resections OR "radical resection" OR "radical resections" OR "lung resection" OR "lung resections" OR "pulmonary resection" OR "pulmonary resections" OR "lung surgery" OR "pulmonary surgery" OR thoracotomy OR thoracotomies OR thoracoscopic OR thoracoscopy OR VATS OR robotic OR "robot-assisted" OR "robotic-assisted" OR RATS OR "lymph node dissection" OR "lymph node dissections" OR "mediastinal lymph node dissection" OR "mediastinal lymph node dissections" OR "mediastinal lymphadenectomy" OR lymphadenectomy OR "lymph node sampling")

#4 #1 AND #2 AND #3

Records retrieved (n):112

Cochrane Library

Stepwise search strategy:

#1 "lung cancer":ti,ab,kw OR "lung cancers":ti,ab,kw OR "lung carcinoma":ti,ab,kw OR "lung carcinomas":ti,ab,kw OR "lung neoplasm":ti,ab,kw OR "lung neoplasms":ti,ab,kw OR "lung tumor":ti,ab,kw OR "lung tumors":ti,ab,kw OR "lung tumour":ti,ab,kw OR "lung tumours":ti,ab,kw OR "pulmonary carcinoma":ti,ab,kw OR "pulmonary carcinomas":ti,ab,kw OR "pulmonary neoplasm":ti,ab,kw OR "pulmonary neoplasms":ti,ab,kw OR "pulmonary tumor":ti,ab,kw OR "pulmonary tumors":ti,ab,kw OR "pulmonary tumour":ti,ab,kw OR "pulmonary tumours":ti,ab,kw OR "non small cell lung cancer":ti,ab,kw OR NSCLC:ti,ab,kw OR "small cell lung cancer":ti,ab,kw OR SCLC:ti,ab,kw OR "lung adenocarcinoma":ti,ab,kw OR "lung squamous cell carcinoma":ti,ab,kw

#2 chylothorax:ti,ab,kw OR chylothoraces:ti,ab,kw OR "chylous pleural effusion":ti,ab,kw OR "chylous effusion":ti,ab,kw OR "chyle leak":ti,ab,kw OR "chyle leakage":ti,ab,kw OR "chylous leak":ti,ab,kw OR "chylous leakage":ti,ab,kw OR "chylous fistula":ti,ab,kw OR "chyle fistula":ti,ab,kw OR chylorrhea:ti,ab,kw OR chylorrhoea:ti,ab,kw OR "thoracic duct injury":ti,ab,kw OR "thoracic duct injuries":ti,ab,kw OR "thoracic duct leak":ti,ab,kw OR "thoracic duct leakage":ti,ab,kw OR "thoracic duct fistula":ti,ab,kw

#3 lobectomy:ti,ab,kw OR lobectomies:ti,ab,kw OR "pulmonary lobectomy":ti,ab,kw OR "lung lobectomy":ti,ab,kw OR pneumonectomy:ti,ab,kw OR pneumonectomies:ti,ab,kw OR segmentectomy:ti,ab,kw OR segmentectomies:ti,ab,kw OR "wedge resection":ti,ab,kw OR "wedge resections":ti,ab,kw OR resection:ti,ab,kw OR resections:ti,ab,kw OR "radical resection":ti,ab,kw OR "radical resections":ti,ab,kw OR "lung resection":ti,ab,kw OR "lung resections":ti,ab,kw OR "pulmonary resection":ti,ab,kw OR "pulmonary resections":ti,ab,kw OR "lung surgery":ti,ab,kw OR "pulmonary surgery":ti,ab,kw OR thoracotomy:ti,ab,kw OR thoracotomies:ti,ab,kw OR thoracoscopic:ti,ab,kw OR thoracoscopy:ti,ab,kw OR VATS:ti,ab,kw OR

robotic:ti,ab,kw OR "robot assisted":ti,ab,kw OR "robotic assisted":ti,ab,kw OR RATS:ti,ab,kw OR "lymph node dissection":ti,ab,kw OR "lymph node dissections":ti,ab,kw OR "mediastinal lymph node dissection":ti,ab,kw OR "mediastinal lymph node dissections":ti,ab,kw OR "mediastinal lymphadenectomy":ti,ab,kw OR lymphadenectomy:ti,ab,kw OR "lymph node sampling":ti,ab,kw

#4 #1 AND #2 AND #3

Records retrieved (n):23

Scopus

Stepwise search strategy:

#1 TITLE-ABS-KEY(("lung cancer" OR "lung cancers" OR "lung carcinoma" OR "lung carcinomas" OR "lung neoplasm" OR "lung neoplasms" OR "lung tumor" OR "lung tumors" OR "lung tumour" OR "lung tumours" OR "pulmonary carcinoma" OR "pulmonary carcinomas" OR "pulmonary neoplasm" OR "pulmonary neoplasms" OR "pulmonary tumor" OR "pulmonary tumors" OR "pulmonary tumour" OR "pulmonary tumours" OR "non-small cell lung cancer" OR "non-small-cell lung cancer" OR NSCLC OR "small cell lung cancer" OR "small-cell lung cancer" OR SCLC OR "lung adenocarcinoma" OR "lung squamous cell carcinoma"))

#2 TITLE(chylothorax OR chylothoraces OR "chylous pleural effusion" OR "chyle leak" OR "chyle leakage" OR "chylous leak" OR "chylous leakage" OR "chylous fistula" OR "chyle fistula" OR chylorrhea OR chylorrhoea OR "thoracic duct leak" OR "thoracic duct leakage") OR TITLE-ABS-KEY("postoperative chylothorax" OR "post-operative chylothorax" OR "chylothorax after" OR "chylothorax following" OR "chylothorax complicating" OR "chylothorax complicated" OR "chyle leak after" OR "chyle leakage after" OR "chylous leak after" OR "chylous leakage after")

#3 TITLE-ABS-KEY(lobectomy OR lobectomies OR "pulmonary lobectomy" OR "lung lobectomy" OR pneumonectomy OR pneumonectomies OR segmentectomy OR segmentectomies OR "wedge resection" OR "wedge resections" OR "lung resection" OR "lung resections" OR "pulmonary resection" OR "pulmonary resections" OR "lung cancer surgery" OR "lung cancer surgeries" OR "mediastinal lymph node dissection" OR "mediastinal lymph node dissections" OR "mediastinal lymphadenectomy" OR "lymph node sampling")

#4 #1 AND #2 AND #3

Records retrieved (n):124

Supplementary Table S4. Detailed treatment strategies and additional clinical outcomes of postoperative chylothorax

Study	No. of cases analyzed for management outcomes, n	Non-surgical treatment, n/N	Non-surgical treatment success, n/N (%)	Surgical or interventional treatment, n/N (%)	Final treatment outcome and mortality	Other clinical outcomes
Akin et al. (2012) (19)	26	26/26; NPO + TPN, supplemented by talc pleurodesis via chest tube	19/26 (73.1)	Surgery: 7/26 (26.9); reoperation via thoracotomy, leak suturing or en bloc ligation	Chylothorax control: 25/26 (96.2); mortality: 1/26 (3.8), due to sepsis and multiple organ failure	Conservative success group: mean daily drainage 380 mL, time to chyle cessation 11 d (4–20); surgery group: mean daily drainage 1157 mL/d, mean duration of conservative treatment before surgery 6 d
Bollen et al. (1993) (20)	2	2/2; specific regimen NR	2/2 (100.0)	Surgery: 0/2 (0.0)	Mortality: 0/2 (0.0)	NR
Bryant et al. (2014) (21)	41	41/41; NPO + subcutaneous somatostatin, switched to MCT diet after meeting criteria	37/41 (90.2)	Surgery: 4/41 (9.8); pleurodesis and clipping of lymphatic leak area	Surgery-related mortality: 0/41 (0.0)	Time to diagnosis: postoperative median 3 d; length of hospital stay: 6 d (4–14) in the non-surgical success group and 9 d (6–31) in the group converted to surgery after treatment failure
Cho et al. (2014) (22)	67	67/67; NPO + TPN (46 cases) or low-LCT diet (21 cases); pleurodesis for dietary treatment failure	65/67 (97.0)	Surgery: 2/67 (3.0); procedure NR	Pleurodesis success: 27/27; pleurodesis-related deaths: 0 cases	Length of hospital stay: median 12 d (5–28); time from dietary treatment to tube removal: 12.5 ± 4.9 d (n = 65); time from pleurodesis to tube removal: 4.5 ± 2.5 d
Dong et al. (2022) (23)	37	37/37; NPO + TPN + continuous closed thoracic drainage, albumin infusion if necessary; intrathoracic pleurodesis for ineffective conservative treatment	32/37 (86.5)	Surgery: 5/37 (13.5); VATS thoracic duct ligation	Chylothorax control in the surgery group: 5/5 (100.0)	Surgery group versus non-surgical group: higher mean daily drainage by 777 mL; total drainage 8609.2 ± 4680.3 mL versus 4911.2 ± 3925.5 mL; total hospital stay 20.0 ± 11.0 d versus 30.0 ± 11.1 d; postoperative hospital stay 10.6 ± 7.4 d versus 17.1 ± 9.6 d; chest tube duration 8.2 ± 2.7 d versus 12.3 ± 6.8 d
Fang et al. (2019) (24)	22	22/22; low-fat, high-carbohydrate, high-protein diet, with maintained chest drainage; intravenous nutrition, albumin, somatostatin, electrolyte supplementation, and intrathoracic pleurodesis if necessary	21/22 (95.5)	Surgery: 1/22 (4.5); thoracoscopic ligation of the main thoracic duct	Chylothorax control: 22/22 (100.0); mortality: 0/22 (0.0)	Onset time: mostly postoperative 1–7 d; daily drainage: 300–1050 mL/d (n = 21); chest tube removal/discharge after conservative treatment: 7–18 d (n = 21)
Ji et al. (2024) (26)	108 (including 79 in the low-fat diet group)	108/108; low-fat diet in 79 cases (fat ≤ 5 g/d), TPN in 29 cases	71/79 (89.9)	Surgery: 8/79 (10.1; low-fat diet group); procedure NR	Mortality: 0/79 (0.0; low-fat diet group); recurrence after reoperation: 0/8 (0.0)	Low-fat diet group: adverse prognosis 12/79 (15.2)
Kamtam et al. (2026) (27)	1110	NR	NR	Surgery: 237/1110 (21.4); procedure NR	30-d operative mortality: 15 cases (1.4%)	30-d outcomes: major complications 396/1110 (35.7); median postoperative length of stay 8 d (5–13)
Kanzaki et al. (2003) (28)	6	6/6; continuous chest drainage, NPO, central venous nutrition (CVN); MCT diet added in 3 cases	4/6 (66.7)	Surgery: 2/6 (33.3); VATS ligation of the injured thoracic duct or branches	Chylothorax control: 6/6 (100.0); in-hospital mortality: 0/6 (0.0)	Onset time: postoperative 1–2 d; chest tube duration: 11–133 d, mean 41.3 ± 44.6 d; resumption of a regular diet: postoperative 18–24 d in the conservative group and postoperative 10–11 d in the VATS group; postoperative hospital stay: 24 d in 2 conservative-treatment patients without pyothorax; 12–13 d in the VATS group
Kutlu et al. (2003) (29)	7	7/7; NPO + TPN, switched to a fat-free diet on postoperative day 7 if leakage volume was < 500 mL/d	5/7 (71.4)	Surgery: 2/7 (28.6); re-thoracotomy, fistula ligation reinforced with surrounding tissue	Chylothorax control: 7/7 (100.0); mortality: 1/7 (14.3), due to pneumonia	Time to diagnosis: postoperative 1–4 d; fistula closure time among conservative successes: postoperative 4–17 d; time to re-thoracotomy: postoperative day 5 and day 6
Le Pimpec-Barthes et al. (2002) (30)	25	25/25; chest tube drainage or thoracentesis; fat restriction with MCT substitution, with maintenance of nutrition and electrolyte balance	19/25 (76.0)	Surgery: 6/25 (24.0); thoracic duct ligation, leak suturing, or fibrin glue	Mortality: 1/25 (4.0), due to ARDS	Total chyle volume, drainage duration, and number of thoracenteses were reported in the original article for the overall 26 cases of chylothorax after pulmonary resection and were not separately reported for lung cancer cases: total chyle volume 1.9–27.9 L, mean 7.9 L; drainage duration after lobectomy 4–19 d, mean 10.5 d; number of thoracenteses after pneumonectomy 2–9, mean 4.25

Study	No. of cases analyzed for management outcomes, n	Non-surgical treatment, n/N	Non-surgical treatment success, n/N (%)	Surgical or interventional treatment, n/N (%)	Final treatment outcome and mortality	Other clinical outcomes
Liu et al. (2014) (32)	20	20/20; NPO + parenteral nutrition; some patients received a fat-free/low-fat diet	15/20 (75.0)	Surgery: 5/20 (25.0); VATS reoperation, leak clipping or thoracic duct ligation, with pleurodesis in some cases	Chylothorax control: 20/20 (100.0); mortality: 0/20 (0.0)	Time to diagnosis: postoperative 1–5 d; mean daily drainage: overall 261.4 mL/d, conservative group 204 mL/d, reoperation group 433 mL/d; NPO + parenteral nutrition in conservative group 5.1 d (0–17), chylothorax resolution 6 d (4–14); chest tube removal in reoperation group 10 d (5–13); length of stay: overall 19.3 d, conservative group 17.5 d, reoperation group 24.8 d
Liu et al. (2022) (33)	24	24/24; initial low-fat, high-protein diet; chest tube clamping, chemical pleurodesis, or octreotide if necessary	21/24 (87.5)	Surgery: 2/24 (8.3); thoracic duct suturing/ligation	Chylothorax control: 23/24 (95.8); mortality: 1/24 (4.2), due to cardiopulmonary failure	NR
Petrella et al. (2020) (34)	30	30/30; NPO + TPN; switched to a low-fat diet (fat < 10 g/d) after drainage became clear for ≥ 24 h and drainage volume was < 800 mL/d	20/30 (66.7)	Surgery: 10/30 (33.3); thoracic duct ligation, with possible clipping of lymphatic leak point	Chylothorax control: 30/30 (100.0); 30-d mortality: 1/30 (3.3), due to postoperative acute hemorrhagic pseudocolytis	Time to diagnosis: postoperative 3 d (2–29); pleural fluid TG: 547 mg/dL (253–767); chyle output during conservative treatment: 734 mL/d (450–975); time to reoperation: 15 d (8–17)
Shimizu et al. (2002) (35)	26 (treatment-strategy analysis cohort; 27 cases occurred in total)	26/26; NPO + TPN; OK-432 pleurodesis if necessary	21/26 (80.8)	Surgery: 5/26 (19.2); procedure NR	Chylothorax control: 26/26 (100.0); mortality: 0/26 (0.0)	Time to diagnosis: postoperative 2 d (1–3); time to resumption of a regular diet in the conservative success group: 8 d (4–35); time to reoperation: 14 d (5–35); other complications: 4/26 (15.4)
Son et al. (2025) (6)	21	21/21; NPO + TPN (24–48 h), then switched to a fat-free/low-fat diet; pleurodesis if necessary	20/21 (95.2)	Surgery: 1/21 (4.8); thoracic duct ligation	30-d mortality: 0/21 (0.0)	Chest tube duration: 14.0 d (11.0–17.0); postoperative hospital stay: 16.0 d (14.0–21.0)
Takuwa et al. (2013) (36)	37	37/37; low-fat diet (fat < 10 g/d); OK-432 pleurodesis if necessary	31/37 (83.8)	Surgery: 6/37 (16.2); video-assisted thoracic duct ligation, with leak suturing/TachoComb coverage in some cases	Chylothorax control: 37/37 (100.0); 30-d and in-hospital mortality: 0/37 (0.0)	Time to resumption of a regular diet in the conservative success group: 10 d (5–27); time to chest tube removal: 10 d (5–20) in the conservative success group and 13 d (6–21) in the surgery group; discharge time in the surgery group: 18 d (14–33); complications: 6 cases
Uchida et al. (2016) (37)	50	50/50; low-fat diet (fat < 20 g/d); NPO or OK-432 pleurodesis if necessary	41/50 (82.0)	Surgery: 9/50 (18.0); ligation of thoracic duct leak point or en bloc ligation	Chylothorax control: 50/50 (100.0); 30-d and in-hospital mortality: 0/50 (0.0)	Time to diagnosis: most cases diagnosed by POD 2 (range, POD 1–9); chest tube removal time in the conservative group: 5.5 d (3–11); time to reoperation: 5.5 d after diagnosis (3–12); Time from diagnosis to discharge in the surgery group: 9.5 d (7–19); recurrence after reoperation: 0/9 (0.0)
Wan et al. (2009) (38)	17	17/17; low-fat, high-carbohydrate, high-protein diet; chest drainage; intravenous nutritional support; intrathoracic Sapylin injection	16/17 (94.1)	Surgery: 1/17 (5.9); thoracic re-entry through the original incision, with ligation of the proximal and distal ends of the fistula	Chylothorax control: 17/17 (100.0)	Onset time of chylothorax: postoperative 3–7 d; daily drainage: 300–1200 mL; time to resolution in the conservative success group: 7–12 d; timing of surgery: 10 d after diagnosis
Wang et al. (2019) (40)	4	4/4; specific regimen NR	3/4 (75.0)	Surgery: 1/4 (25.0); VATS thoracic duct ligation	Mortality: 0/4 (0.0)	Chest tube removal time among conservative successes: postoperative 12 d (10–16)
Yang et al. (2013) (42)	3	3/3; specific regimen NR	3/3 (100.0)	Surgery: 0/3 (0.0)	Chylothorax control: 3/3 (100.0)	NR
Yang et al. (2022) (43)	21 (true chylothorax)	21/21; conservative treatment, with adequate drainage, nutritional support, and pleurodesis in 13/21	13/21 (61.9)	Surgery: 8/21 (38.1); right-sided VATS thoracic duct clipping	NR	Pre-treatment drainage volume: 821.19 ± 247.82 mL/d; post-treatment drainage volume: 462.14 ± 145.96 mL/d; postoperative drainage duration: 9.09 ± 3.05 d; length of stay: 17.67 ± 2.74 d
Yasuura et al. (2022) (5)	37	37/37; low-fat diet (fat restriction 10–30 g/d); OK-432 pleurodesis if necessary (5/37)	35/37 (94.6)	Surgery: 2/37 (5.4); supradiaphragmatic thoracic duct ligation with TachoComb coverage of the leak point	Chylothorax control: 37/37 (100.0); 30-d mortality: 0/37 (0.0)	Drainage volume on day 1 after low-fat diet: < 500 mL in 28/37 and ≥ 500 mL in 9/37 (including ≥ 1000 mL/d in 2/37); chest tube removal time: 4 d (1–12) in the < 500 mL group, 5 d (5–9) in the ≥ 500 mL conservative group, and 6.5 d (5–8) in the surgery group; other complications: 5 cases

Study	No. of cases analyzed for management outcomes, n	Non-surgical treatment, n/N	Non-surgical treatment success, n/N (%)	Surgical or interventional treatment, n/N (%)	Final treatment outcome and mortality	Other clinical outcomes
Zhao et al. (2003) (44)	12	10/12; thoracentesis drainage or prolonged chest tube retention	10/12 (83.3)	Surgery: 2/12 (16.7); thoracic duct ligation	Chylothorax control: 12/12 (100.0)	Time to diagnosis: within postoperative 4 d in 4 cases, 5–10 d in 7 cases, and after 2 weeks in 1 case; thoracentesis drainage: 8 cases, mean 4.5 times, mean chyle loss 1875 mL

Note: This table includes only studies that reported management strategies or clinical outcomes of postoperative chylothorax. When denominators differed by outcome, the denominator used is reported in the corresponding cell. Pleurodesis was classified as non-surgical treatment when performed without a surgical or interventional procedure; when used as part of a surgical or interventional procedure, it was described within the corresponding treatment strategy. Missing mortality information or mortality reported as NR indicates that mortality was not reported in the original article and should not be interpreted as zero deaths. For Le Pimpec-Barthes et al. (2002)(30) and Shimizu et al. (2002)(35), the denominators for management outcomes differed from the incidence denominators; the management-outcome denominators reported in the corresponding cells were used in the pooled analyses. Kamtam et al. (2026) and Zhao et al. (2003) were summarized descriptively but were excluded from the pooled analysis of subsequent surgical or interventional treatment because the treatment sequence was not clearly reported.

Abbreviations: NR = not reported; NPO = nil per os/no oral intake; TPN = total parenteral nutrition; CVN = central venous nutrition; MCT = medium-chain triglyceride; LCT = long-chain triglyceride; VATS = video-assisted thoracoscopic surgery; TG = triglyceride; POD = postoperative day; d = days; h = hours; ARDS = acute respiratory distress syndrome; OK-432 = Picibanil; TachoComb = fibrinogen-coated collagen patch/hemostatic material.
