# Supplementary material for: Incidence and management outcomes of chylothorax after pulmonary resection for primary lung cancer: a systematic review and meta-analysis
Source: Front Oncol. 2026 Sep 15;16:1910973. doi: 10.3389/fonc.2026.1910973 (PMC13619438; doi:10.3389/fonc.2026.1910973)
Supplement: Supplementary file 2 [file DataSheet2.pdf]

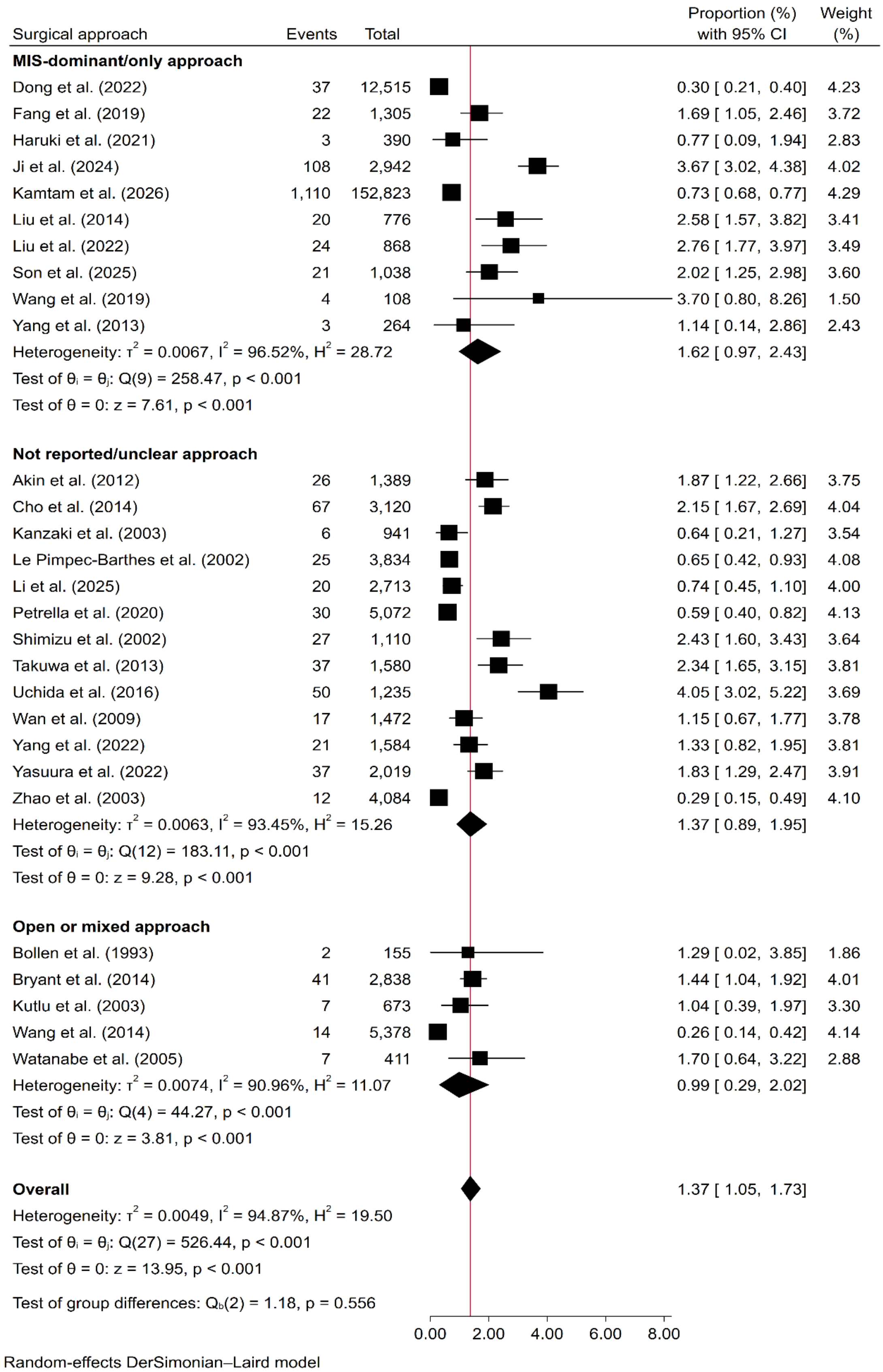

Supplementary Figure S1. Forest plot of postoperative chylothorax incidence stratified by surgical approach.

Diagnostic definition	Events	Total	Proportion (%) with 95% CI	Weight (%)
**Not reported/unclear definition**				
Bollen et al. (1993)	2	155	1.29 [0.02, 3.85]	1.86
Haruki et al. (2021)	3	390	0.77 [0.09, 1.94]	2.83
Li et al. (2025)	20	2,713	0.74 [0.45, 1.10]	4.00
Wang et al. (2014)	14	5,378	0.26 [0.14, 0.42]	4.14
Watanabe et al. (2005)	7	411	1.70 [0.64, 3.22]	2.88
Yang et al. (2013)	3	264	1.14 [0.14, 2.86]	2.43
Heterogeneity: $\tau^2 = 0.0032$, $I^2 = 77.48\%$, $H^2 = 4.44$			0.75 [0.30, 1.36]	
Test of $\theta_i = \theta_j$: Q(5) = 22.20, p < 0.001				
Test of $\theta = 0$: z = 4.65, p < 0.001				
**Other reported definition**				
Fang et al. (2019)	22	1,305	1.69 [1.05, 2.46]	3.72
Kamtam et al. (2026)	1,110	152,823	0.73 [0.68, 0.77]	4.29
Kanzaki et al. (2003)	6	941	0.64 [0.21, 1.27]	3.54
Liu et al. (2022)	24	868	2.76 [1.77, 3.97]	3.49
Wan et al. (2009)	17	1,472	1.15 [0.67, 1.77]	3.78
Wang et al. (2019)	4	108	3.70 [0.80, 8.26]	1.50
Zhao et al. (2003)	12	4,084	0.29 [0.15, 0.49]	4.10
Heterogeneity: $\tau^2 = 0.0032$, $I^2 = 89.91\%$, $H^2 = 9.91$			1.07 [0.61, 1.64]	
Test of $\theta_i = \theta_j$: Q(6) = 59.47, p < 0.001				
Test of $\theta = 0$: z = 7.05, p < 0.001				
**TG-threshold definition**				
Akin et al. (2012)	26	1,389	1.87 [1.22, 2.66]	3.75
Bryant et al. (2014)	41	2,838	1.44 [1.04, 1.92]	4.01
Cho et al. (2014)	67	3,120	2.15 [1.67, 2.69]	4.04
Dong et al. (2022)	37	12,515	0.30 [0.21, 0.40]	4.23
Ji et al. (2024)	108	2,942	3.67 [3.02, 4.38]	4.02
Kutlu et al. (2003)	7	673	1.04 [0.39, 1.97]	3.30
Le Pimpec-Barthes et al. (2002)	25	3,834	0.65 [0.42, 0.93]	4.08
Liu et al. (2014)	20	776	2.58 [1.57, 3.82]	3.41
Petrella et al. (2020)	30	5,072	0.59 [0.40, 0.82]	4.13
Shimizu et al. (2002)	27	1,110	2.43 [1.60, 3.43]	3.64
Son et al. (2025)	21	1,038	2.02 [1.25, 2.98]	3.60
Takuwa et al. (2013)	37	1,580	2.34 [1.65, 3.15]	3.81
Uchida et al. (2016)	50	1,235	4.05 [3.02, 5.22]	3.69
Yang et al. (2022)	21	1,584	1.33 [0.82, 1.95]	3.81
Yasuura et al. (2022)	37	2,019	1.83 [1.29, 2.47]	3.91
Heterogeneity: $\tau^2 = 0.0100$, $I^2 = 96.27\%$, $H^2 = 26.79$			1.72 [1.10, 2.47]	
Test of $\theta_i = \theta_j$: Q(14) = 375.01, p < 0.001				
Test of $\theta = 0$: z = 9.01, p < 0.001				
**Overall**			1.37 [1.05, 1.73]	
Heterogeneity: $\tau^2 = 0.0049$, $I^2 = 94.87\%$, $H^2 = 19.50$				
Test of $\theta_i = \theta_j$: Q(27) = 526.44, p < 0.001				
Test of $\theta = 0$: z = 13.95, p < 0.001				
Test of group differences: $Q_b(2) = 4.12$, p = 0.128				

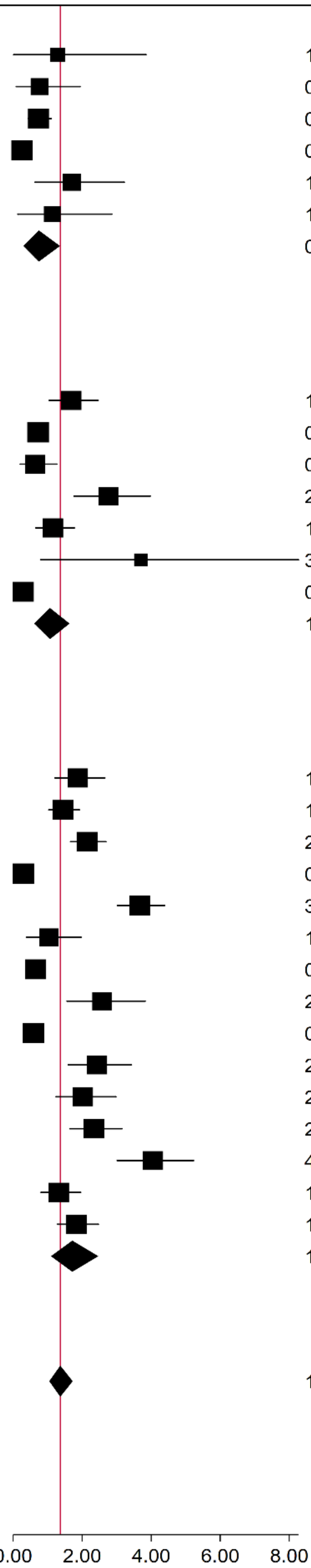

Random-effects DerSimonian–Laird model

Supplementary Figure S2. Forest plot of postoperative chylothorax incidence stratified by diagnostic criteria.

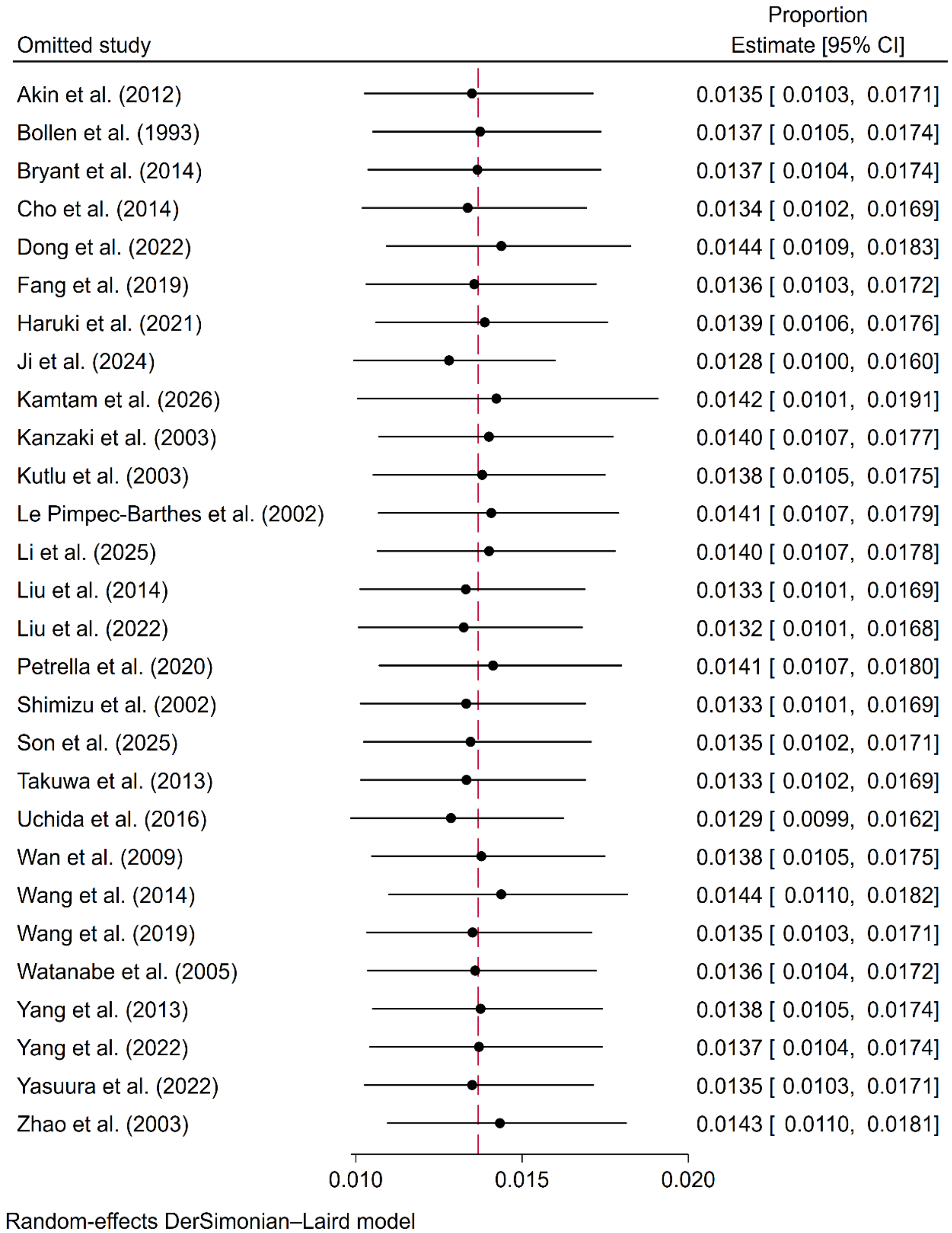

Supplementary Figure S3. Leave-one-out sensitivity analysis for postoperative chylothorax incidence.

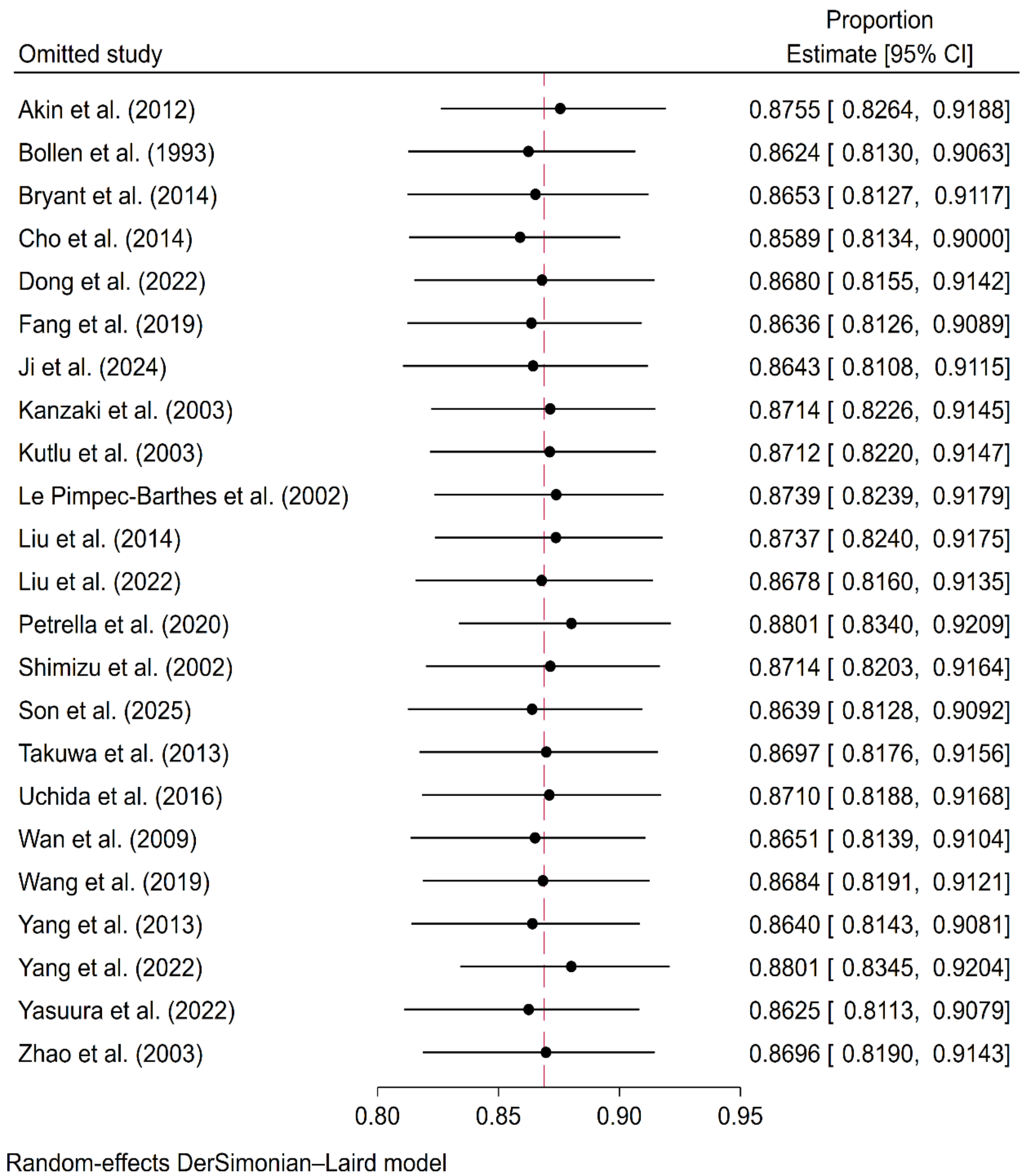

Supplementary Figure S4. Leave-one-out sensitivity analysis for the proportion of patients successfully treated non-surgically.

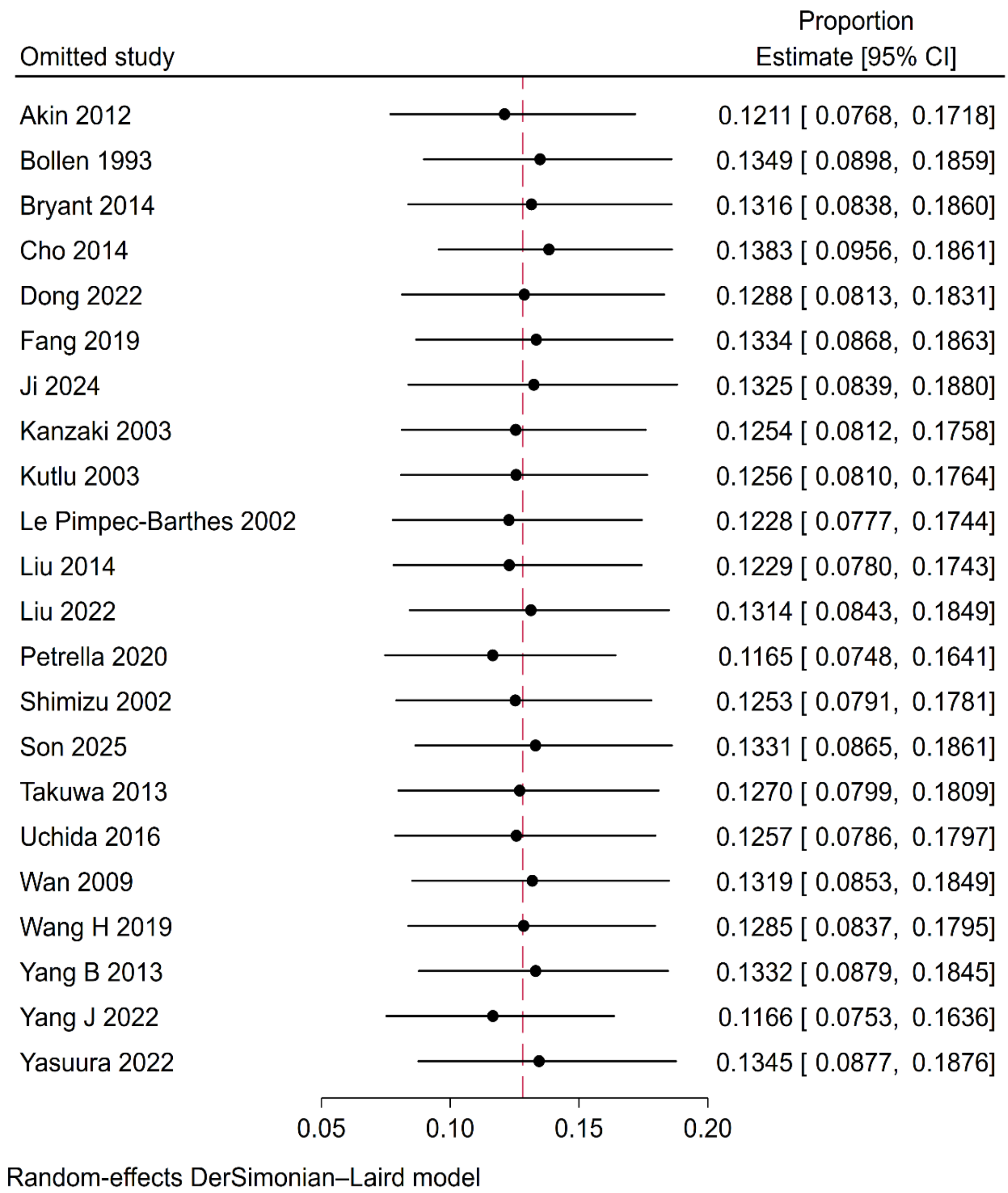

Supplementary Figure S5. Leave-one-out sensitivity analysis for the proportion of patients receiving subsequent surgical or interventional treatment.

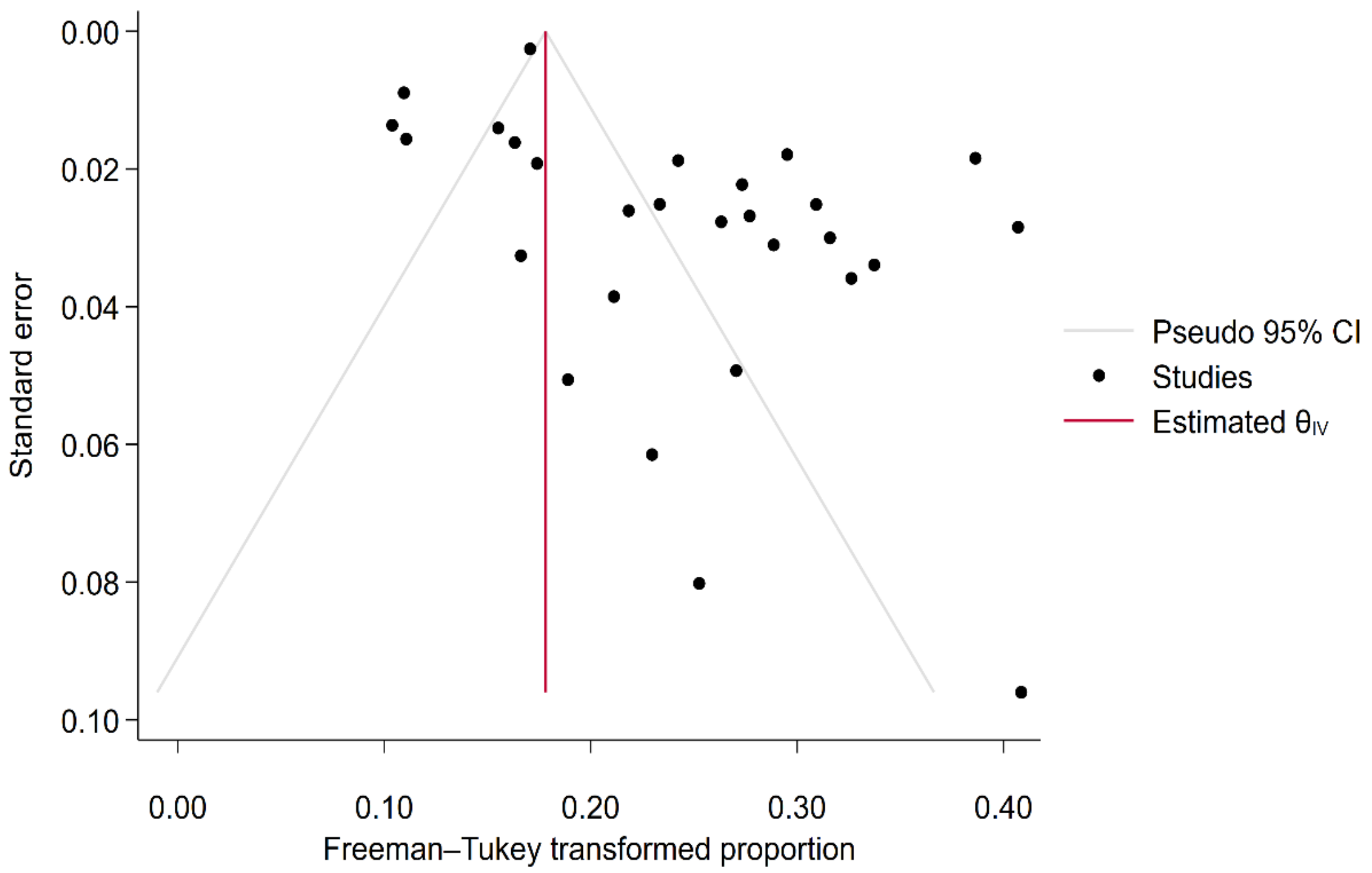

Supplementary Figure S6. Funnel plot for postoperative chylothorax incidence.

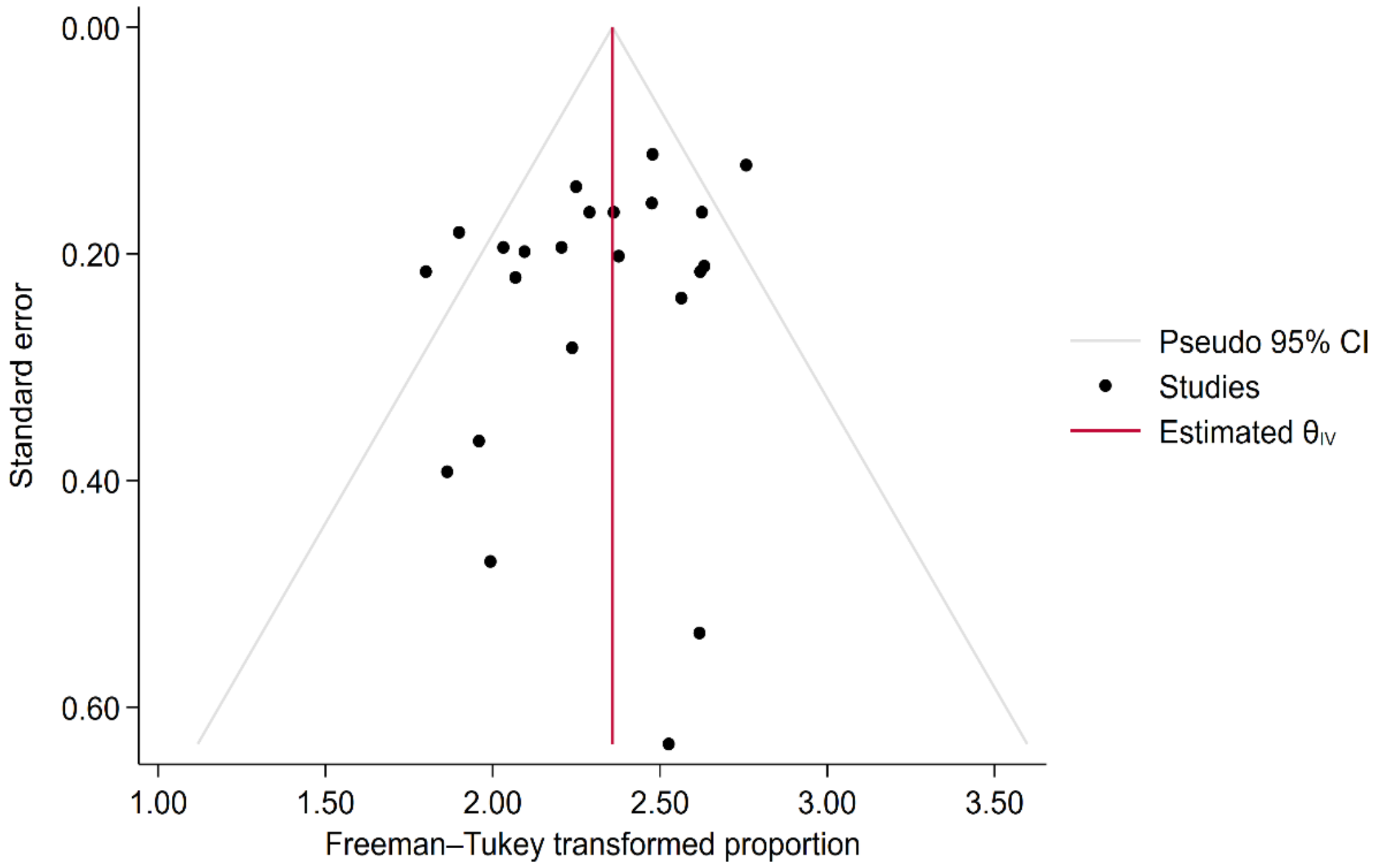

Supplementary Figure S7. Funnel plot for the proportion of patients successfully treated non-surgically.

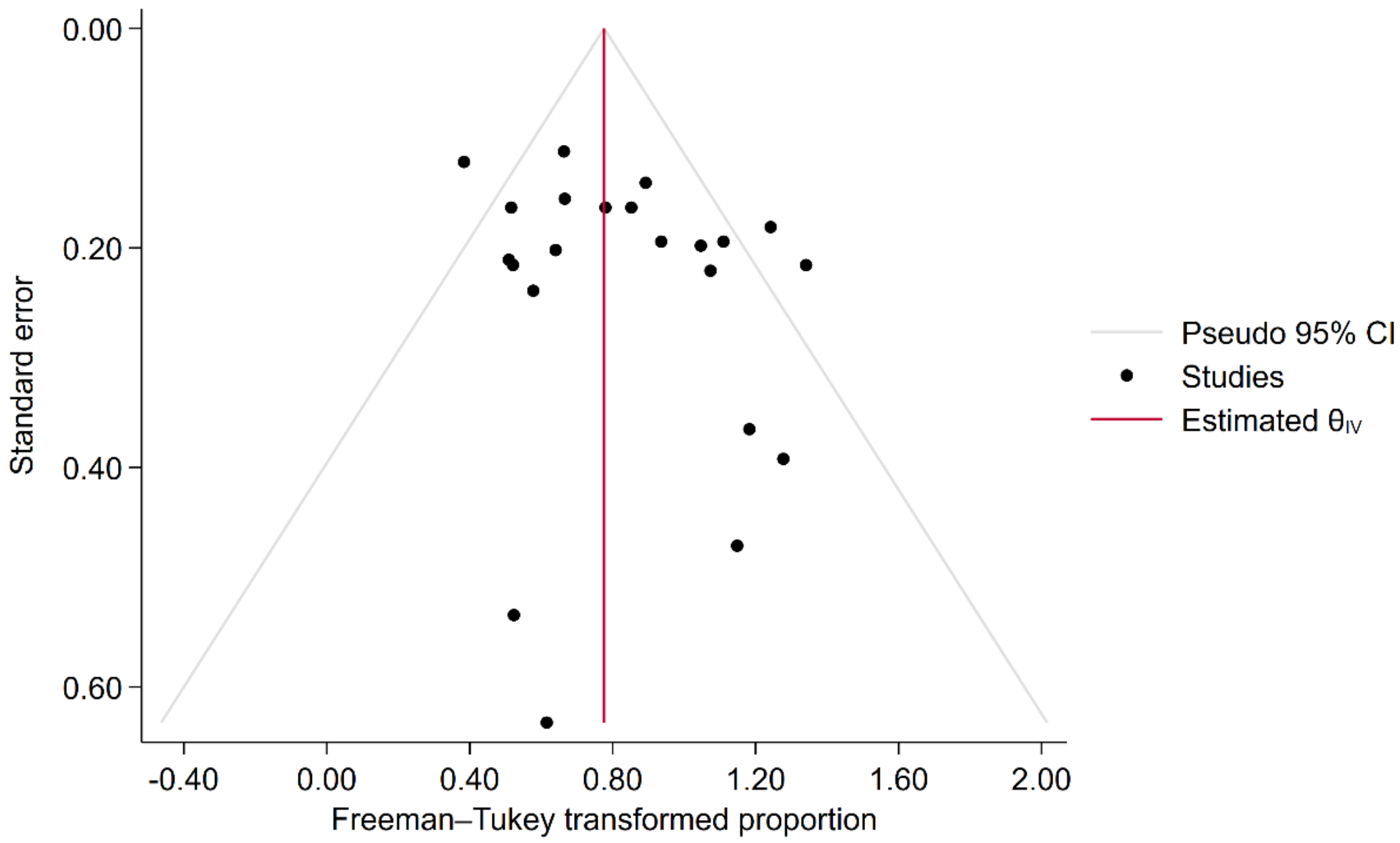

Supplementary Figure S8. Funnel plot for the proportion of patients receiving subsequent surgical or interventional treatment.
